# Modulation of the gut-heart axis by exercise in diabetic cardiomyopathy: Microbial mechanisms and clinical implications

**DOI:** 10.1016/j.isci.2026.115490

**Published:** 2026-03-26

**Authors:** Jun Chen, Zhiwei Fan, Yuan Li, Rui Duan, Binghong Gao

**Affiliations:** 1 Faculty of Health Sciences and Sports, Macao Polytechnic University, Macao 999078, China; 2 SCNU-MPU Joint Lab for Sports Science, School of Physical Education and Sports Science, South China Normal University, Guangzhou, Guangdong 510006, China; 3 School of Athletic Performance, Shanghai University of Sport, Shanghai 200438, China

**Keywords:** Health sciences, Medicine, Medical specialty, Internal medicine, Cardiovascular medicine, Endocrinology

## Abstract

Diabetic cardiomyopathy (DCM) is a common complication of diabetes, a leading cause of diabetes-related mortality. Growing evidence indicates a strong association between gut microbiota dysbiosis and the development and progression of DCM. This dysbiosis disrupts intestinal barrier integrity and alters metabolite profiles, including short-chain fatty acids, bile acids, trimethylamine-N-oxide, lipopolysaccharide, branched-chain amino acids, hydrogen sulfide, and tryptophan metabolites, thereby promoting inflammation, oxidative stress, and metabolic dysfunction through the gut-heart axis, ultimately contributing to the pathogenesis of DCM. Studies have shown that exercise reshapes gut microbiota composition, enhances intestinal barrier function, and modulates the production of microbial metabolites in individuals with diabetes. Exercise has been recommended as a first-line intervention for patients with DCM. However, whether exercise prevents and treats DCM through gut microbiota regulation and the underlying mechanisms of this interaction remain largely unclear. This review summarizes current evidence linking exercise, gut microbiota, and DCM, emphasizing the potential role of gut microbiota in mediating the cardioprotective effects of exercise in diabetes. Understanding how exercise modulates the gut-heart axis may open new avenues for microbiota-based strategies to prevent and manage DCM.

## Graphical abstract

**Figure:**
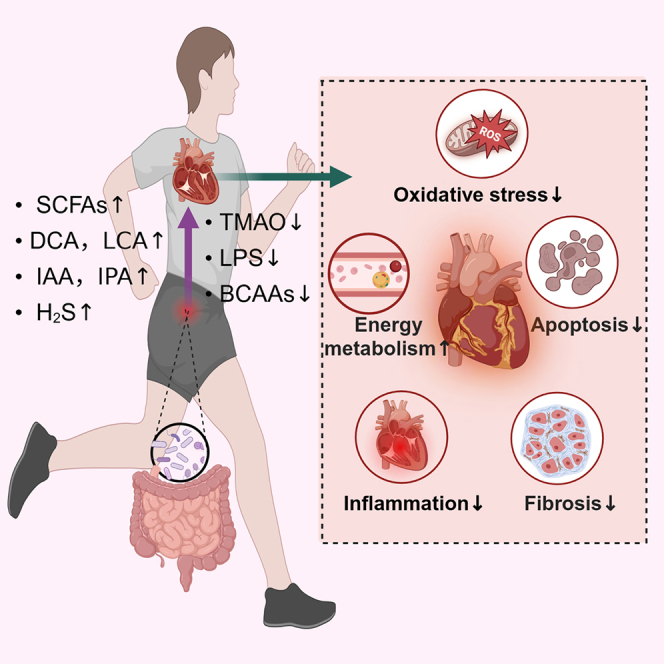

## Introduction

Diabetes has emerged as a major global health challenge, with its prevalence continuing to rise and projected to reach 783 million cases by 2045.^1^ Diabetic cardiomyopathy (DCM) is a severe complication of diabetes, defined by structural and functional myocardial abnormalities occurring in the absence of other known causes of cardiac injury, such as hypertension, coronary artery disease, or valvular disease.^2^ The prevalence of DCM is estimated at ∼14.5% in patients with type 1 diabetes (T1D)^3^ and up to 30% in those with type 2 diabetes (T2D).^4^ Without timely intervention, DCM can progress to chronic heart failure, markedly increasing mortality risk.^5^ Despite its growing clinical burden, DCM remains inadequately addressed by current therapeutic strategies. Existing pharmacological treatments—such as calcium channel blockers and angiotensin-converting enzyme inhibitors—provide partial symptomatic relief but fail to reverse disease progression and are often accompanied by adverse effect.^6^ Thus, exploring novel intervention strategies for DCM is of urgent clinical importance.

In this context, exercise has emerged as a clinically indispensable, non-pharmacological strategy for the management of DCM.^7^ Unlike pharmacological agents that typically target single molecular pathways, exercise exerts broad, system-level benefits, including improved glycemic control, attenuation of myocardial inflammation and oxidative stress, enhanced mitochondrial function, and delayed ventricular remodeling.^8^^,^^9^^,^^10^ Importantly, these pleiotropic effects uniquely position exercise to overcome key limitations of current drug-based approaches, highlighting its irreplaceable role in the comprehensive management of DCM.^11^^,^^12^

Over the past decades, the gut microbiota has emerged as a pivotal regulator of host metabolic, immune, and cardiovascular functions.^13^ Increasing evidence indicates that gut microbiota dysbiosis—characterized by reduced microbial diversity and altered microbial composition and function—is implicated in the pathogenesis of multiple cardiometabolic disorders, including obesity, T2D, and heart failure.^14^ Recent studies further reveal that gut microbial dysbiosis and associated shifts in microbial metabolites exert profound effects on cardiac health, forming the conceptual basis of the gut-heart axis.^15^^,^^16^ In DCM, gut microbiota dysbiosis is common and is accompanied by impaired intestinal barrier function, along with disturbances in key microbial metabolites, such as short-chain fatty acids (SCFAs), trimethylamine-N-oxide (TMAO), and bile acids (BAs).^17^^,^^18^^,^^19^ These alterations promote myocardial inflammation, oxidative stress, and metabolic dysfunction, ultimately contributing to DCM progression.^15^ Therefore, targeting the gut microbiota has emerged as a promising strategy for preventing and treating DCM.

Notably, exercise is increasingly recognized as an effective modulator of the gut microbiota, capable of restoring microbial diversity and promoting the growth of beneficial commensal taxa, thereby ameliorating diabetes-associated gut dysbiosis.^20^^,^^21^ These findings suggest that exercise-induced improvements in the gut microbiota may promote cardiac health via the gut-heart axis. However, despite these parallel lines of evidence, a critical knowledge gap remains: most studies examine exercise, gut microbiota alterations, or DCM outcomes in isolation, and the mechanistic role of exercise-induced gut microbiota remodeling in DCM has not been systematically synthesized or critically evaluated.

In this review, we comprehensively synthesize current evidence on the relationship between DCM and the gut microbiota, delineate the modulatory effects of exercise on gut microbial composition, and highlight potential mechanisms through which exercise may prevent and treat DCM via the gut-heart axis. This review aims to provide a theoretical foundation and novel insights for exercise-based interventions in DCM.

## Pathophysiology of DCM

The pathophysiological mechanisms linking diabetes to cardiomyopathy remain incompletely understood. Current evidence indicates that insulin resistance, impaired myocardial energy metabolism, oxidative stress, mitochondrial dysfunction, defective autophagy, excessive apoptosis, inflammation, and myocardial fibrosis act in concert to drive the onset and progression of DCM.^22^^,^^23^^,^^24^^,^^25^

Chronic hyperglycemia accelerates the formation of advanced glycation end-products (AGEs), which induce extracellular matrix (ECM) crosslinking and activate receptor for AGE (RAGE) signaling.^6^ The resulting activation of nuclear factor kappa B (NF-κB) and mitogen-activated protein kinase (MAPK) pathways amplifies inflammatory responses and upregulates the expression of pro-inflammatory mediators, such as intercellular adhesion molecule-1 (ICAM-1), vascular cell adhesion molecule-1 (VCAM-1), tumor necrosis factor-α (TNF-α), and interleukin-6 (IL-6).^26^ These inflammatory mediators promote cardiomyocyte hypertrophy and ECM remodeling, ultimately exacerbating adverse ventricular remodeling and fibrosis.^27^

Furthermore, impaired insulin signaling markedly reduces glucose uptake and utilization in cardiomyocytes, compelling the diabetic heart to rely excessively on fatty acid β-oxidation.^28^ Although fatty acids provide abundant acetyl-CoA, their oxidation is oxygen intensive and metabolically inefficient, markedly increasing reactive oxygen species (ROS) generation and exacerbating oxidative stress and mitochondrial dysfunction.^29^ In diabetes, nutrient overload enhances CD36-mediated fatty acid uptake and promotes accumulation of toxic lipid intermediates, such as ceramides and diacylglycerols, thereby aggravating lipotoxicity, impairing mitochondrial membrane potential, and activating pro-apoptotic signaling pathways.^30^^,^^31^ Protein misfolding combined with lipid excess triggers endoplasmic reticulum (ER) stress, which activates ‌C/EBP homologous protein‌(CHOP)-mediated apoptotic signaling and further contributes to cardiomyocyte loss.^32^ Under normal conditions, cardiomyocytes counteract metabolic stress through autophagy, which removes damaged mitochondria.^33^ However, energy surplus suppresses mechanistic target of rapamycin (mTOR)-mediated autophagy, leading to accumulation of dysfunctional mitochondria, enhanced apoptosis, and ultimately cardiomyocyte death and tissue injury.^32^^,^^34^

The impact of diabetes on cardiac health extends beyond cardiomyocytes and profoundly affects the cardiac microvascular system. Coronary microvascular dysfunction represents a critical early event in DCM pathogenesis. Chronic exposure to hyperglycemia and inflammatory cytokines (e.g., IL-6, TNF-α) enhances oxidative stress in endothelial cells and reduces endothelial nitric oxide synthase (eNOS) phosphorylation, thereby diminishing nitric oxide (NO) bioavailability.^35^ Reduced NO production impairs vasodilation, increases microvascular resistance, and leads to impaired coronary microvascular reserve and progressive diastolic dysfunction.^36^^,^^37^

Beyond these mechanisms, calcium homeostasis imbalance,^38^ pyroptosis,^39^ ferroptosis,^40^ and cardiac autonomic neuropathy^41^ have also been implicated in diabetic cardiac dysfunction. Importantly, emerging evidence highlights the gut-heart axis as a previously underappreciated contributor to DCM pathogenesis. Gut microbial dysbiosis and altered microbial metabolites, such as SCFAs, TMAO, and BAs, can modulate inflammation, oxidative stress, mitochondrial function, and metabolic flexibility, thereby converging on key pathways involved in DCM progression.^42^^,^^43^^,^^44^ This expanded mechanistic framework provides a theoretical basis for exploring targeted modulation of the gut microbiota as a novel strategy for DCM prevention and treatment.

## Gut microbiota and DCM

### Overview of the gut microbiota

The gut microbiota is a vast and complex microbial community residing in the human intestine, composed primarily of bacteria, fungi, and viruses.^45^ These microorganisms outnumber human cells by approximately 10-fold, and their collective genome encodes nearly 150 times more genes than the human genome.^46^ Consequently, the gut microbiota is often referred to as the human “second genome”. In healthy individuals, *Firmicutes* and *Bacteroidetes* account for more than 90% of the total intestinal bacterial population, and their abundance ratio (F/B) serves as a common indicator of gut microbiota dysbiosis. Other dominant phyla include *Actinobacteria* and *Proteobacteria*.^47^

Humans and the gut microbiota have co-evolved.^48^ The host provides nutrients and an appropriate habitat for the gut microbiota, which in turn regulate diverse physiological functions, including immune system development and maturation,^49^ nutrient metabolism and absorption,^50^ and neuroendocrine and metabolic.^51^ Under normal physiological conditions, the gut microbiota maintain a dynamic equilibrium.^13^ However, this equilibrium can be disturbed by factors, such as antibiotic use,^52^ unhealthy dietary habits,^53^ sedentary behavior,^54^ psychological stress,^55^ and infections,^56^ thereby inducing gut microbiota dysbiosis. Extensive research has shown that gut microbiota dysbiosis is implicated in the pathogenesis of multiple metabolic diseases, including obesity, T2D, and cardiovascular disorders.^57^^,^^58^^,^^59^ Therefore, targeting the gut microbiota has emerged as a promising strategy for preventing and treating these conditions.

### Dysbiosis of gut microbiota and DCM

DCM is a complex cardiometabolic disorder influenced by multiple factors, including both genetic and environmental components. Among these factors, long-term unhealthy dietary habits represent a major risk factor for DCM, as they directly alter gut microbiota composition and induce microbial dysbiosis.^60^ Furthermore, impaired cardiac function can lead to intestinal hypoxia, hypercapnia, and elevated norepinephrine levels, all of which further disrupt the gut microbial community and exacerbate gut microecological imbalance.^61^

Compared with healthy controls, patients with DCM exhibit a marked reduction in gut microbial diversity, accompanied by an increased proportion of *Actinobacteria* and *Proteobacteria* and a decreased proportion of *Firmicutes*.^62^ Notably, *Firmicutes* abundance has been reported to correlate positively with left ventricular ejection fraction in patients with T2D, whereas its reduction is associated with an increased risk of left ventricular hypertrophy.^17^ In addition, the abundances of *Faecalibacterium*, *Ruminococcaceae*, *Roseburia*, and particularly the probiotic genus *Bifidobacterium* are decreased, whereas the opportunistic pathobiont *Escherichia*, which is closely linked to insulin resistance, hyperglycemia, and increased cardiovascular risk, is enriched.^18^^,^^62^^,^^63^ At the species level, *Faecalibacterium prausnitzii*, a butyrate-producing bacterium with anti-inflammatory properties and a key role in maintaining intestinal barrier integrity, was markedly reduced in patients with DCM, whereas *Ruminococcus gnavus*, a pro-inflammatory species, was significantly increased.^18^

Importantly, these compositional alterations are closely associated with impaired intestinal barrier function, which represents a critical physical interface linking gut dysbiosis to cardiac injury.^64^ Beneficial commensal bacteria, such as *Faecalibacterium*, *Roseburia*, and *Bifidobacterium*, contribute to epithelial integrity by promoting the expression of tight junction proteins, including zonula occludens-1 (ZO-1), occludin, and claudins.^65^ Their depletion in DCM is therefore associated with downregulation of tight junction proteins and increased intestinal permeability.^43^^,^^66^ Conversely, expansion of *Proteobacteria* and *Escherichia* species exacerbates epithelial damage and disrupts tight junction architecture, thereby promoting a “leaky gut” phenotype.^67^^,^^68^

As a consequence of intestinal barrier dysfunction, microbial components such as lipopolysaccharide (LPS) can translocate from the gut lumen into the systemic circulation, resulting in chronic low-grade endotoxemia.^69^ Elevated circulating LPS activates Toll-like receptor 4 (TLR4)-dependent inflammatory signaling pathways, thereby amplifying systemic inflammatory responses.^70^ This inflammatory milieu contributes to myocardial macrophage activation, oxidative stress, and fibrosis, ultimately exacerbating adverse cardiac remodeling and functional decline in DCM.^27^ Thus, intestinal barrier impairment and low-grade systemic inflammation constitute key upstream mechanisms linking gut microbiota dysbiosis to myocardial injury in DCM.

Consistent with human data, most DCM animal models also display reduced microbial diversity, increased abundances of *Escherichia coli*, and decreased levels of SCFA-producing bacteria, including *Roseburia*, *Faecalibaculum*, and *Bifidobacterium*.^43^^,^^44^^,^^71^^,^^72^^,^^73^ Interestingly, increased abundances of *Lactobacillus*, *Bacteroides*, and *Akkermansia*, genera typically considered beneficial, have also been observed in DCM animal models.^71^^,^^72^^,^^73^ A plausible explanation is that the increased abundance of these genera may reflect pathological compensatory responses. For instance, the genus *Lactobacillus* exhibits substantial heterogeneity, and many of its species proliferate more readily under DCM-associated intestinal conditions, such as elevated luminal glucose levels and increased acidity.^74^^,^^75^ *Akkermansia*, particularly *Akkermansia muciniphila*, is a classic mucin-degrading bacterium; damage to the intestinal mucosal barrier often increases mucin synthesis and secretion, providing substrates that promote its compensatory expansion.^76^ Similarly, *Bacteroides* can utilize host-derived mucins and dietary polysaccharides, which are often elevated in metabolic environments associated with high-fat diets, thereby supporting its expansion.^77^^,^^78^ Hence, their enrichment likely reflects gut dysbiosis-driven adaptive responses rather than a purely beneficial signature; however, this interpretation requires further validation.

In parallel, functional alterations in the gut microbiota have also been observed in patients with DCM. For example, compared with healthy individuals, the gut microbiota of patients with DCM exhibit increased abundances of microbial genes involved in the phosphotransferase system, along with reduced gene pathways related to amino acid and nucleotide sugar biosynthesis. More importantly, microbial genes involved in the synthesis of LPS, tryptophan metabolites, and TMAO are markedly upregulated in DCM.^18^ Both LPS and TMAO exhibit pro-inflammatory activity and induce systemic inflammation, which promotes myocardial fibrosis and impairs cardiac contractile function.^79^ In contrast, the gene encoding butyryl-CoA:acetate CoA-transferase, the key enzyme for butyrate synthesis, shows reduced abundance in DCM.^18^ This reduction in butyrate availability may impair myocardial fatty acid oxidation and metabolic flexibility, thereby forcing a greater reliance on less efficient glycolytic pathways and ultimately contributing to an energy-deficient metabolic state.^80^ Furthermore, DCM animal models also exhibit reduced microbial capacity for branched-chain amino acid (BCAA) degradation,^81^ along with BA metabolic disturbances.^82^ Studies have shown that elevated BCAAs suppress mitochondrial autophagy and fatty acid oxidation in cardiomyocytes, leading to cardiac dysfunction.^81^ Meanwhile, disturbed BA metabolism promotes myocardial lipid deposition and fibrosis by disrupting farnesoid X receptor (FXR) and the membrane-bound Takeda G-protein-coupled receptor 5 (TGR5) signaling (discussed in detail in the following text).^82^^,^^83^

Recent evidence indicates that interventions targeting the restoration of gut microbial homeostasis, including polyphenol supplementation,^43^ traditional Chinese medicine,^71^^,^^72^^,^^73^ and fecal microbiota transplantation (FMT) from healthy donors,^81^ can attenuate cardiac dysfunction and remodeling in DCM models, further supporting the mechanistic link between gut microbiota and DCM.

In conclusion, patients with DCM exhibit pronounced gut microbial dysbiosis characterized by reduced diversity, diminished abundance of beneficial bacteria, increased levels of opportunistic pathobionts, and functional alterations (Table 1). However, most current studies primarily focus on dominant phyla and genera, whereas alterations and functional roles of low abundance and rare taxa remain largely unexplored. Moreover, the dynamic changes in gut microbiota composition and function during the progression from diabetes to DCM remain unclear, and future longitudinal studies are needed to elucidate these trajectories. Furthermore, gut microbiota composition in DCM is also influenced by host-related factors, such as age, sex, diabetes duration, dietary patterns, and concomitant pharmacological treatments (e.g., metformin, sodium-glucose cotransporter 2 inhibitors),^17^^,^^84^^,^^85^^,^^86^ which are not consistently controlled across existing studies and may contribute to inter-study heterogeneity. Importantly, much of the current mechanistic evidence is derived from animal models of DCM, most commonly induced by streptozotocin administration or extreme dietary interventions.^87^ While these models provide valuable mechanistic insights, they do not fully recapitulate the chronic, heterogeneous, and multifactorial nature of human DCM nor do they account for key interspecies differences in immune responses, cardiac energy utilization, and gut microbiota composition.^15^^,^^88^^,^^89^ Consequently, caution is warranted when extrapolating findings from these models to human disease. To strengthen translational relevance, future studies should integrate well-phenotyped human cohorts with advanced experimental approaches, such as humanized gut microbiota models generated by transplanting fecal microbiota from patients with DCM into germ-free animals, to better delineate causal relationships and microbiome-driven mechanisms in DCM.

**Table 1:** Changes in gut microbiota composition in human or animal models with DCM

Study	Study subjects/country	Microbiome analysis	Abundance changes
Chen et al.^62^	30 patients with DCM, 40 patients with T2D, and 30 healthy volunteers from China	metagenomic sequencing	*Proteobacteria, Actinobacteria*, *Lactobacillus mucosae*, *Escherichia coli*, and *Bacteroides fragilis* ↑; *Firmicutes*, *Bifidobacterium*, *Roseburia*, *Ruminococcus*, *Trueperella*, and *Megamonas*↓
Cui et al.^18^	53 patients with chronic heart failure (including 28 with DCM) and 41 healthy volunteers from China	metagenomic sequencing	*Alistipes*, Oscillibacte, *Ruminococcus gnavus* and *Olsenella*↑; *Bifidobacterium*, *Faecalibacterium Blautia*, *Lactobacillus*, *Candidatus Brocadia*, and *Faecalicoccus*↓
Yang et al.^71^	DCM mice	16S rRNA sequencing	*Akkermansia* and *Escherichia coli*↑; *Muribaculaceae-unclassified*, *Ligilactobacillus*, and *Lachnospiraceae*↓
Zhu et al.^43^	DCM mice	16S rRNA sequencing	*Bacilli*, *Roseburia*, *Bifidobacterium*, and *Faecalibaculum*↓
Huang et al.^72^	DCM mice	16S rRNA sequencing	*Lactobacillus*, *Bacteroides*, *Alloprevotella*, and *Alistipes*↑; *Lachnospiraceae*, *Ruminococcaceae*, *Eubacterium*, and *Desulfovibrio*↓
Fang et al.^73^	DCM mice	16S rRNA sequencing	*Lactobacillus*, *Oscillospira*, and *Bacteroides*↑; *Ruminococcus*, *Lachnospiraceae-Clostridium*, and *Desulfovibrio*↓
Khalaf et al.^44^	DCM mice	qPCR	*Bacteroides*, *Escherichia coli*, *Fusobacterium*, *Clostridium*, and *Providencia*↑; *Bifidobacterium*, *Lactobacillus*, and *Enterococcus faecium*↓

## Effects of exercise on gut microbiota in patients with T2D

### Effects of exercise on gut microbiota composition and diversity in patients with T2D

Exercise acts as an independent regulator of gut health and gut microbiota homeostasis.^90^ Regular exercise increases both α-diversity (within-sample diversity) and β-diversity (between-sample diversity) of the gut microbiota, resulting in a richer and more balanced microbial composition.^91^^,^^92^ This effect appears to be particularly pronounced in individuals with low baseline microbial diversity, such as those with T2D.^93^^,^^94^^,^^95^ Adequate microbial diversity is essential for maintaining host-microbiota symbiosis and intestinal ecological stability, both of which are fundamental to gut health.^96^ Beyond increasing diversity, exercise also restores compositional imbalances of the gut microbiota associated with T2D. Evidence from clinical and preclinical studies indicates that moderate- to high-intensity regular exercise reduces the F/B ratio. In addition, exercise increases the abundance of beneficial bacteria—particularly SCFA-producing taxa, such as *A. muciniphila*, *Bifidobacterium*, and *Roseburia*—and decreases proinflammatory bacteria, such as *Enterobacteriaceae*, *Clostridium*, and *Blautia*.^94^^,^^95^^,^^97^^,^^98^^,^^99^^,^^100^^,^^101^ These alterations suggest attenuation of gut-derived proinflammatory signaling and enrichment of microbial taxa that support host metabolic health. Indeed, exercise-induced shifts in the gut microbiota are frequently associated with improved insulin sensitivity and reduced systemic inflammation in individuals with T2D.^97^^,^^98^

Although most studies indicate that regular exercise induces beneficial changes in the gut microbiota, these effects appear to be strongly modulated by baseline gut microbial features and host body composition. For example, a recent study in men with prediabetes revealed substantial interindividual variability in exercise efficacy, with approximately 30% of participants exhibiting a non-response, defined as no detectable changes in gut microbiota or insulin resistance after training. Further analysis showed that exercise non-responders exhibited marked increases in *Bacteroides xylanisolvens* and *Alistipes shahii*, taxa associated with harmful microbial metabolites. In contrast, responders exhibited an increased abundance of *Streptococcus oralis*, along with microbial functional profiles characterized by enhanced SCFA synthesis and BCAA degradation. Notably, FMT from exercise responders, but not non-responders, improved insulin resistance in obese mice, indicating that baseline gut microbiota plays a central role in driving exercise responsiveness.^99^ Allen et al. reported that 6 weeks of endurance training increased the abundance of SCFA-producing bacterial genes and taxa in lean individuals, whereas no significant changes were observed in those with obesity. Importantly, these exercise-induced microbial shifts rapidly disappeared after training cessation, indicating that the gut microbiota is highly dependent on sustained exercise stimuli.^102^ Therefore, regular exercise is likely essential for sustaining long-term, stable, and beneficial shifts in the gut microbiota.

In summary, these findings indicate that physically active patients with T2D exhibit a more diverse and metabolically favorable gut microbiota, which may contribute to improved glycemic control and reduced inflammation (Table 2). Future research should clarify which baseline gut microbiota profiles determine the therapeutic response to exercise, and how this relationship is modified by key host- and treatment-related factors, including diet, medication use, age, sex, and diabetes duration. Such insights will facilitate the development of targeted strategies to modulate baseline microbiota, such as supplementation with specific probiotics, to enhance exercise efficacy in non-responders.

**Table 2:** Effects of exercise on gut microbiota in T2D patients and animal models

Study	Subjects	Intervention protocol	Abundance changes
Motiani et al.^97^	patients with T2D	AE, 60% VO_2max_, 5 times/week, 60 min/session, 2 weeks	*Bacteroidetes*, *Veillonella*, *Faecalibacterium↑*; *Clostridium*, *Blautia*↓
Pasini et al.^98^	patients with T2D	AE + RE + FE,3 times/week, 90 min/session, 6 months	*Candida albicans*, *Mycetes* spp.↓
Torquati et al.^94^	patients with T2D	MICT: 55%–69% HR_max_, 52.5 min/session, 4 times/week, 8 weeks; HIIT: 85%–95% HR_max_, 26 min/session, 3 times/week, 8 weeks	MICT: *Bifidobacterium*, *A. muciniphila*, *Lachnospira eligens*, *Enterococcus* spp., *Clostridium Cluster*↑ HIIT: *Oscillospira*, *Erysipelotrichales*↑
Liu et al.^99^	male patients with prediabetes	AE + RE, 80%–95% HR_max_, 3 times/week, 70 min/session, 12 weeks	*Roseburia*, *Anaerotruncus Phascolarctobacterium*, *Bacteroides vulgatus*↑; *Bilophila wadsworthia*, *Allistipes putredinis*↓
Yang et al.^100^	T2D mice	AE, 5 times/week, 60 min/session, 8 weeks	*Bacteroidetes*, *Lactobacillus*, *Alloprevotella*↑; *Firmicutes*, *Proteobacteria*, *Dubosiella*, *Helicobacter*↓
Han et al.^101^	T2D rat	MICT: 17 m/min, 60 min/session, 7 times/week, 8 weeks; HIIT: 25 m/min, 40 min/session, 7 times/week, 8 weeks	MICT: *Allobaculum*, *Coprococcus*↑; HITT: *Rothia*, *Ruminococcus*↓
Lambert et al.^95^	T2D mice	AE, 2.87 m/min, 60 min/session,3 times/week, 6 weeks	*Lactobacillus*, *Clostridium*↑; *Enterobacteriaceae*, *Methanobrevibacter*↓

AE, aerobic exercise; RE, resistance exercise; FE, flexibility exercise; VO_2max_, maximal oxygen uptake; HR_max_, maximum heart rate; MICT, moderate-intensity continuous training; HIIT, high-intensity interval training.

### Possible mechanisms of exercise-regulated gut microbiota in patients with T2D

It is well established that exercise induces hormetic stress, thereby triggering a cascade of adaptive physiological responses. As an integral component of the human superorganism, the gut microbiota also responds dynamically to these physiological stressors.^20^ Studies have shown that exercise enhances intestinal peristalsis and consequently shortens gastrointestinal transit time.^103^ This shift favors the colonization of bacteria with shorter replication cycles, such as *Bifidobacterium* and other beneficial taxa, while limiting the expansion of bacteria with longer replication cycles, many of which are potentially pathogenic, thereby optimizing the overall gut microbial composition.^104^^,^^105^

In addition, exercise-induced shifts in gut microbiota composition may be closely linked to alterations in the host internal physiological environment. Under physiological conditions, the colon exhibits a “low-oxygen outer, anaerobic inner” gradient, in which obligate anaerobes—many of which are beneficial bacteria—predominate.^106^ Exercise increases mesenteric blood flow and improves mucosal perfusion, which may indirectly support the maintenance of anaerobic luminal conditions and limit the overgrowth of facultative anaerobic pathogens such as *E. coli*.^107^ At the same time, exercise induces systemic lactate accumulation, which may subsequently lower intestinal luminal pH.^108^ In general, acidification of the intestinal environment favors the colonization of beneficial bacteria such as *Lactobacillus* and *Bifidobacterium*, thereby helping maintain gut microbial homeostasis.^109^^,^^110^

Collectively, these studies suggest that the effects of exercise on the gut microbiota may arise from exercise-induced stress responses and concomitant alterations in the host physiological environment. However, evidence in patients with T2D remains limited, and further well-designed clinical studies are needed to validate these mechanistic pathways.

### Association between exercise prescription and gut microbiota in patients with T2D

Although exercise induces beneficial alterations in the gut microbiota, the enriched microbial genera and species vary considerably depending on the type, intensity, duration, and frequency of exercise. For instance, individuals engaged in long-term endurance training show an enrichment of SCFA-producing bacteria, such as *Bifidobacterium* and *Bacteroides*, which contribute to optimizing the gut environment and enhancing nutrient absorption.^111^ In contrast, resistance training tends to increase bacteria involved in protein metabolism, such as *Sutterella* and *Haemophilus*, which may support protein digestion and muscle repair.^111^ These findings suggest that different exercise types may selectively promote specific metabolically functional microbes, shaping unique gut microbial profiles. Furthermore, several studies propose that combined aerobic and resistance training may exert synergistic effects on gut microbiota remodeling by integrating the benefits of both modalities. Specifically, aerobic exercise improves intestinal perfusion and mucosal oxygenation, whereas resistance exercise induces lactate production that modulates the gut microenvironment.^93^^,^^112^ However, whether combined exercise confers superior benefits compared with single exercise in patients with T2D remains to be further elucidated.

At the level of exercise intensity, Torquati et al. compared the effects of high-intensity interval training (HIIT) and moderate-intensity continuous training (MICT) on the gut microbiota of patients with T2D. They found that MICT increased the relative abundance of butyrate-producing bacteria, including *Bifidobacterium* and *A. muciniphila*, whereas HIIT predominantly enriched *Oscillospira* and other butyrate-producing bacteria. Overall, MICT promoted a greater variety of butyrate-producing taxa. Additionally, the MICT group exhibited greater activity in pathways associated with pyruvate metabolism and cell wall biogenesis, suggesting that exercise intensity distinctly affects gut microbial composition and metabolic function.^94^ Similarly, Han et al. found in T2D rats that MICT enhanced microbial homogeneity and specific beneficial species enrichment more effectively than HIIT, suggesting a better gut microbial response to MICT.^101^ This may be because MICT provides a more stable metabolic and physiological environment, such as sustained energy metabolism, lower oxidative stress levels, and improved intestinal perfusion, which supports the growth of beneficial butyrate-producing bacteria and more effectively maintains gut homeostasis.^20^ Although higher-intensity exercise is more likely to induce detectable microbiota remodeling, excessive intensity—especially when combined with prolonged duration—may transiently reduce commensal bacteria (e.g., *Faecalibacterium prausnitzii* and *Eubacterium rectale*) and increase potentially unfavorable taxa (e.g., *Streptococcus* spp., *Haemophilus* spp., and, in some athlete cohorts, *Proteobacteria/Enterobacteriaceae*), suggesting that excessive physiological stress can perturb gut microbial homeostasis.^113^^,^^114^^,^^115^

In terms of exercise frequency and duration, McFadzean et al. reported that, compared with individuals exercising 1–2 times per week, those engaging in physical activity 3–5 times per week showed a more pronounced increase in gut microbial α-diversity and in the abundance of *Firmicutes*—particularly *Faecalibacterium*,^116^ and Evans et al. found in high-fat diet-induced obese mice that extending exercise duration from 6 to 12 weeks progressively increased α-diversity and further reduced the F/B ratio.^117^ According to the review by Boytar et al., exercising 2–3 times per week appears sufficient to induce changes in β-diversity and genus-level abundance, whereas exercising 4–5 times per week and intervention durations exceeding 6–8 weeks are more consistently associated with increases in α-diversity and stable enrichment of beneficial taxa, such as *Bifidobacterium*.^112^ Similarly, exercise durations of 30–90 min are most often associated with favorable microbial changes, whereas sessions exceeding 90 min induce stronger perturbations that may selectively favor taxa, such as *Veillonella*, *Akkermansia*, or *Streptococcus*, likely reflecting greater metabolic stress, transient hypoxia, and alterations in intestinal permeability. In contrast, Cronin et al. reported that aerobic exercise sessions lasting 18–32 min did not alter gut microbiota diversity and overall composition in sedentary adults, suggesting that exercise stimuli may need to exceed a certain duration threshold to overcome baseline microbial resilience and induce measurable shifts in the gut microbial community.^118^ Taken together, available evidence suggests that this duration threshold may be approximately 30 min, although further confirmation in well-controlled trials is warranted. Collectively, these findings indicate that the regulatory effects of exercise on the gut microbiota are highly dependent on both exercise frequency and duration, reinforcing the concept of a dose-dependent microbial response to exercise.

In conclusion, current evidence supports the existence of a preliminary “exercise dose-gut microbiota response” pattern that is likely characterized by an inverted U-shaped relationship. The heterogeneous effects of different exercise prescriptions on gut microbiota composition and abundance are likely driven by distinct physiological and ecological pressures imposed on the intestinal microenvironment. Exercise parameters such as intensity, duration, and frequency differentially influence intestinal perfusion, oxygen tension, substrate availability, luminal pH, and host immune-endocrine signaling, all of which act as selective forces shaping microbial communities.^20^ However, the optimal exercise type, intensity, frequency, and duration for enhancing microbial diversity and beneficial taxa in patients with T2D remain unclear and warrant further investigation. Moreover, diet should be considered a major confounding factor, as aerobic exercisers often consume more carbohydrates, resistance trainers typically increase protein and fat intake, and higher exercise intensity or frequency is generally accompanied by greater energy intake, all of which can influence gut microbiota composition.^20^ Therefore, future studies should control dietary variables to elucidate the independent effects of different exercise prescriptions on the gut microbiota in individuals with T2D.

## Potential mechanisms by which exercise modulates the gut microbiota to prevent and treat DCM

### Regulation of gut microbiota-derived metabolites

**Figure 1:**
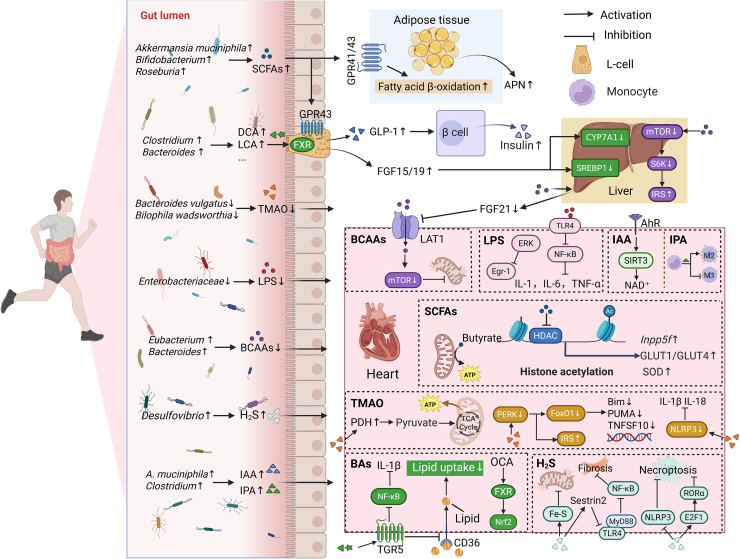
Mechanisms by which exercise modulates gut microbiota-derived metabolites to prevent and treat DCM By reshaping gut microbiota composition, exercise enhances the production of SCFAs, secondary BAs, H_2_S, and indole derivatives, while reducing the generation of TMAO, LPS, and BCAAs. On one hand, these metabolites act on peripheral metabolic organs, such as the gut (e.g., increased GLP-1 and FGF15/19 secretion), liver (e.g., reduced FGF21 secretion), and adipose tissue (e.g., elevated APN production), thereby indirectly improving cardiac metabolic health. On the other hand, these metabolites directly influence the myocardium via the gut-heart axis by suppressing inflammation, oxidative stress, and mitochondrial injury, as well as reducing cardiac lipotoxicity and apoptosis, ultimately contributing to the prevention and amelioration of DCM. GPR41/43, G protein-coupled receptor 41/43; APN, adiponectin; FGF15/19, fibroblast growth factor 15/19; S6K1, ribosomal protein S6 kinase 1; MyD88, myeloid differentiation primary response 88; TCA, tricarboxylic acid cycle. Created in BioRender. Chen, J. (2025) https://BioRender.com/pimqwf7.

Gut microbiota-derived metabolites serve as a crucial link between the gut and the heart. Increasing evidence indicates that gut microbiota-derived metabolites—such as SCFAs, BAs, TMAO, LPS, BCAAs, hydrogen sulfide (H_2_S), and tryptophan metabolites, enter the circulation and function as signaling molecules that interact with distant organs, including the heart, thereby influencing the onset and progression of DCM.^15^ Current evidence shows that exercise regulates the production of these metabolites by reshaping gut microbiota composition, suggesting that microbiota-derived metabolites may serve as key mediators of the cardioprotective effects of exercise in DCM (Figure 1).

#### SCFAs

SCFAs—primarily acetate, propionate, and butyrate—are metabolites generated by the fermentation of dietary fiber and other indigestible carbohydrates by gut microbiota. Most SCFAs are absorbed by colonic epithelial cells, while the remainder enters the systemic circulation via the portal vein or the inferior vena cava pathway.^119^ Compared with healthy individuals, patients with DCM show a significant reduction in SCFA-producing bacteria and lower circulating SCFA levels, which are closely associated with adverse cardiac remodeling and functional impairment.^18^^,^^120^

Studies have shown that exercise increases the abundance of SCFA-producing bacteria in the gut, thereby reversing diabetes-induced decreases in colonic and plasma SCFA levels.^94^ These metabolites regulate metabolic homeostasis, immune function, and epigenetic modification by activating G protein-coupled receptors (e.g., GPR41 and GPR43) or functioning as endogenous inhibitors of histone deacetylase (HDAC), thereby mediating several of the cardiometabolic benefits of exercise.^119^^,^^121^ For example, gut-derived SCFAs activate GPR43 on colonic L cells, promoting glucagon-like peptide-1 (GLP-1) secretion, an incretin that enhances insulin secretion and sensitivity.^122^ In diabetic models, blockade of GPR43 signaling abolishes the insulin-sensitizing effects of exercise, indicating that the SCFA-GPR43 axis is a critical pathway mediating exercise-induced metabolic regulation.^100^ Additionally, SCFAs activate GPR41/43 in adipose tissue, thereby promoting fatty acid β-oxidation and the secretion of adiponectin, an adipokine that enhances fatty acid oxidation and suppresses lipolysis.^123^ These effects reduce circulating free fatty acids and help alleviate cardiac lipotoxicity.

Accumulating evidence suggests that SCFAs also exert direct actions on cardiomyocytes, thereby contributing to the regulation of myocardial energy metabolism. One key mechanism involves the activation of AMP-activated protein kinase (AMPK), a master energy sensor that coordinates glucose and lipid metabolism in cardiomyocytes.^124^ Indeed, SCFAs, particularly butyrate and propionate, have been shown to activate AMPK either directly by increasing the cellular AMP/ATP ratio or indirectly via GPR41/43-mediated signaling.^125^ AMPK activation enhances fatty acid β-oxidation by phosphorylating and inhibiting acetyl-CoA carboxylase, thereby reducing malonyl-CoA levels and relieving inhibition of carnitine palmitoyltransferase-1 (CPT1).^126^ Concurrently, AMPK promotes glucose uptake by enhancing the expression and membrane translocation of glucose transporter 1 (GLUT1) and glucose transporter 4 (GLUT4), ultimately improving myocardial substrate flexibility.^127^^,^^128^ In addition, SCFAs modulate myocardial energy metabolism via peroxisome proliferator-activated receptors (PPARs), particularly PPARα and PPARδ, which are central regulators of mitochondrial fatty acid oxidation in the heart.^129^^,^^130^ SCFAs have been shown to increase the expression and activity of PPARα target genes involved in fatty acid transport and oxidation, including CPT1, medium-chain acyl-CoA dehydrogenase, and acyl-CoA oxidase.^131^ Through activation of PPARs, SCFAs may further enhance mitochondrial oxidative capacity and improve metabolic adaptability under diabetic conditions. These AMPK-PPAR-mediated effects collectively contribute to the restoration of myocardial metabolic flexibility, a hallmark of exercise-induced cardioprotection.

Recent evidence indicates that SCFAs function as efficient alternative energy substrates for the myocardium, and even under heart failure conditions, ATP production from butyrate oxidation remains substantially higher than that generated from ketone bodies.^132^ In high-fat, high-sugar diet-induced metabolic heart disease mouse models, acute supplementation with 4 mmol L^−1^ butyrate increases cardiac ATP synthesis and restores contractile performance.^133^ Therefore, exercise-induced SCFAs may be utilized by cardiomyocytes to enhance myocardial energy homeostasis.

Epigenetic regulation represents another crucial mechanism through which SCFAs, particularly butyrate, exert cardiometabolic protective effects. Acting as an HDAC inhibitor, butyrate enters cardiomyocytes to suppress HDAC activity, thereby increasing histone acetylation. This epigenetic mechanism upregulates cardioprotective genes such as *Inpp5f* and enhances the expression of GLUT 1 and GLUT4, as well as antioxidant enzyme superoxide dismutase (SOD), ultimately attenuating cardiomyocyte apoptosis, oxidative stress, and metabolic dysfunction in diabetic mice.^134^ In addition to histone modification, butyrate may also modulate DNA methylation. In DCM, reduced circulating butyrate levels are associated with hypermethylation of the *HIF3A* promoter, suggesting that butyrate deficiency impair cardiac hypoxia adaptation.^135^ Thus, exercise-induced SCFAs can epigenetically reprogram cardiomyocytes, activating cardioprotective gene networks and promoting metabolic reprogramming in the diabetic heart.

In summary, exercise-induced SCFAs exert cardioprotective effects in DCM by activating GPR41/43 signaling pathways, inhibiting HDAC activity, and serving as myocardial energy substrates, thereby improving metabolic function and reducing oxidative stress and apoptosis in cardiomyocytes. However, whether these endogenous SCFAs can replicate the benefits observed with exogenous supplementation remains unclear, because most exercise-induced SCFAs are rapidly absorbed and metabolized in the colon, resulting in much lower systemic and myocardial exposure.^136^^,^^137^ Thus, although SCFAs may exert biological effects at low concentrations, the cardioprotective relevance of exercise-induced SCFAs remains uncertain and warrants further investigation.

#### BAs

BAs are essential components of bile. Primary BAs, such as cholic acid (CA) and chenodeoxycholic acid (CDCA), are synthesized from cholesterol in the liver and conjugated at the C24 carboxyl group with taurine or glycine. Upon entering the intestine, these conjugated BAs are deconjugated and converted into secondary BAs, including lithocholic acid (LCA) and deoxycholic acid (DCA), by gut bacteria harboring bile salt hydrolase (BSH) activity, such as members of the genera *Clostridium* and *Bacteroides*.^138^ BAs exert biological effects by activating two major receptors: the nuclear receptor FXR and the membrane receptor TGR5. These receptors are widely expressed in the intestine, liver, heart, and vasculature, and together they regulate glucose and lipid metabolism, energy expenditure, and inflammation.^139^ FXR is predominantly activated by CDCA and DCA, whereas TGR5 exhibits higher affinity for LCA and DCA.^140^ In DCM, gut dysbiosis disrupts BA metabolism, leading to reduced total BA levels and an imbalance between primary and secondary BAs—particularly those that preferentially activate FXR and TGR5, such as DCA and LCA. The reduction of these BAs has been linked to diabetic cardiac injury, suggesting that disturbances in BA metabolism may contribute to DCM pathogenesis.^82^^,^^83^

Exercise reshapes the gut microbiota, increasing the abundance of BSH-containing bacteria, such as *Parabacteroides* and *Bacteroides*, and enhancing the deconjugation of bile salts.^141^^,^^142^ This leads to a rebalanced BA pool with elevated levels of free primary BAs (e.g., CDCA) and secondary BAs (e.g., LCA and DCA).^143^^,^^144^ The expanded and modified BA profile can act locally within the intestine to activate FXR and TGR5 in ileal L cells, thereby stimulating GLP-1 secretion and enhancing glucose homeostasis.^145^^,^^146^ Additionally, FXR activation in ileal L cells induces fibroblast growth factor 15/19 (FGF15/19), which subsequently suppresses hepatic sterol regulatory element-binding protein 1 (SREBP1) and cholesterol 7α-hydroxylase (CYP7A1) expression to coordinate lipid and glucose metabolism.^147^ Systemically, these effects contribute to improve glycemic control and lipid profiles, thereby reducing metabolic substrate overload and lipotoxicity that drive DCM.

Exercise not only affects local BA transformation within the intestine but also modulates BA homeostasis at the level of the enterohepatic circulation. Through coordinated regulation of intestinal FXR signaling, FGF15/19 secretion, and hepatic CYP7A1 activity, exercise functionally couples intestinal BA sensing with hepatic BA synthesis, thereby fine-tuning both the size and composition of the circulating BA pool.^148^ This integrative regulation enables exercise to synchronize hepatic BA production with intestinal BA signaling demands, rather than merely altering luminal BA conversion.^149^ In the context of diabetes, such systemic modulation of the enterohepatic circulation may be particularly important for preventing excessive BA synthesis, correcting BA pool imbalance, and alleviating metabolic stress in the diabetic heart.^82^^,^^150^

In addition to improve systemic metabolism, BAs can directly activate FXR and TGR5 receptors in the myocardium to cf. cardioprotection. FXR deficiency markedly aggravates myocardial injury in diabetic mice, characterized by impaired cardiac function, increased fibrosis, and excessive lipid accumulation.^150^ Conversely, treatment with obeticholic acid (OCA), an FXR agonist, alleviates myocardial fibrosis, inflammation, and oxidative stress by upregulating the nuclear factor erythroid 2-related factor 2 (Nrf2)-mediated antioxidant pathway.^83^ Similarly, cardiomyocyte-specific deletion of TGR5 in diabetic mice causes severe cardiac injury with lipid accumulation and contractile dysfunction, whereas supplementation with DCA and LCA activates intestinal TGR5, suppresses CD36-mediated lipid uptake, and thereby mitigates these impairments.^82^ Moreover, activation of the DCA-TGR5 signaling pathway suppresses NF-κB activity and downstream IL-1β expression in cardiomyocytes under hypoxic conditions.^151^ Given the chronic hypoxic and pro-inflammatory milieu of the diabetic myocardium, secondary BA-mediated TGR5 activation may help mitigate hypoxia-induced myocardial inflammation.^152^ These findings suggest that BA-FXR/TGR5 signaling represents an important pathway through which exercise may exert cardioprotective effects.

In summary, by reshaping gut microbial composition and improving BA metabolism, exercise may enhance FXR and TGR5 signaling, thereby ameliorating DCM. However, given the substantial diversity and complexity of both the gut microbiota and the BA pool, elucidating the relationships between specific BAs and specific microbial taxa remains a major challenge. Future research should systematically investigate these interactions to facilitate the precise development of targeted therapeutic strategies for DCM.

#### TMAO

TMAO is another gut microbiota-derived metabolite that serves as a key mediator linking dietary patterns, metabolic disturbances, and cardiovascular diseases.^153^ Dietary nutrients, such as choline, L-carnitine, and betaine are metabolized by intestinal bacteria to generate trimethylamine (TMA), which enters the liver via the portal vein and is rapidly oxidized to TMAO by flavin-containing monooxygenases (FMOs).^154^ Numerous studies have shown that elevated circulating TMAO levels in T2D patients are closely associated with increased risk of adverse cardiovascular outcomes, suggesting that TMAO may represent a potential pathogenic factor in diabetes-related cardiac injury.^155^^,^^156^^,^^157^

Mechanistically, TMAO aggravates cardiac dysfunction primarily by inducing inflammatory responses. Elevated TMAO levels activate the MAPK and NF-κB signaling pathways in vascular smooth muscle and endothelial cells, thereby promoting leukocyte adhesion, enhancing endothelial inflammatory gene expression, and stimulating the release of pro-inflammatory cytokines, including IL-1β, IL-6, and TNF-α.^158^ Additionally, TMAO activates the NLR family pyrin domain-containing protein 3 (NLRP3) inflammasome in cardiac fibroblasts, leading to caspase-1 activation and subsequent release of IL-1β and IL-18.^159^ This persistent cardiovascular low-grade inflammatory response ultimately contributes to myocardial fibrosis, microvascular dysfunction, and impaired cardiac performance.

Beyond its well-recognized pro-inflammatory effects, recent evidence indicates that TMAO also disrupts metabolic homeostasis in cardiomyocytes. In DCM, TMAO binds to the ER stress sensor protein kinase RNA-like ER kinase (PERK) in cardiomyocytes, thereby activating forkhead box protein O1 (FoxO1).^72^ Excessive activation of FoxO1 in the cardiomyocytes downregulates insulin receptor substrate (IRS) expression and disrupts downstream insulin signaling, while simultaneously inducing the transcription of pro-apoptotic genes, such as *Bim*, *PUMA*, and *TNFSF10*.^160^^,^^161^ In addition, TMAO inhibits pyruvate dehydrogenase (PDH) activity, thereby limiting pyruvate entry into the tricarboxylic acid cycle and impairing mitochondrial fatty acid oxidation.^162^^,^^163^ This metabolic inflexibility results in reduced ATP production, lipid accumulation, and heightened susceptibility to contractile dysfunction, thereby exacerbating cardiac injury in the diabetic heart.^164^

Physical exercise can reduce the production of gut microbiota-derived TMAO. In individuals at high risk for T2D, higher levels of physical activity are associated with lower plasma TMAO concentrations.^165^ Moreover, regular exercise decreases the abundance of TMA-producing bacteria in individuals with prediabetes, including *Bilophila wadsworthia* and *Bacteroides vulgatus*, thereby limiting the microbial conversion of dietary choline and carnitine into TMA and reducing hepatic oxidation of TMA to TMAO.^99^ Notably, the cardioprotective effects of exercise may be partly attributable to reduced TMAO generation. For example, in a Western diet-induced obese mouse model, voluntary exercise lowered plasma TMAO levels and reduced myocardial inflammation and fibrosis, whereas exogenous TMAO supplementation blunted these exercise-induced benefits.^166^ Consistently, another study showed that 8 weeks of MICT mitigated TMAO-induced myocardial injury and restored multiple cardiac metabolic pathways, including amino acid metabolism (e.g., alanine and glycine), energy metabolism (tricarboxylic acid cycle), and oxidative stress-related pathways (purine metabolism), suggesting that exercise can counteract the cardiotoxicity of TMAO by preventing TMAO-driven metabolic reprogramming in the heart.^167^ Together, these findings suggest that exercise improve cardiac metabolic health through the gut-heart axis by reducing TMAO production.

#### LPS

LPS is an endotoxin and a defining structural component of the outer membrane of Gram-negative bacteria. In patients with T2D and DCM, the gut exhibits a marked expansion of LPS-enriched Gram-negative bacteria, particularly members of the *Escherichia* family such as *E. coli*.^62^^,^^168^ Upon bacterial death or lysis, LPS is released into the intestinal lumen, where it triggers epithelial apoptosis and oxidative stress, disrupts gut mucosal integrity, and subsequently translocates across the gut barrier into the systemic circulation.^169^ Once in circulation, LPS binds to TLR4 on immune cells as well as on various tissues and organs, including the heart, liver, and adipose tissue. This interaction activates downstream NF-κB pathway, inducing the release of pro-inflammatory cytokines, such as TNF-α, IL-1β, and IL-6, thereby creating a chronic low-grade inflammatory milieu that promotes to the development and progression of DCM.^79^ Moreover, gut microbiota-derived LPS further aggravates diabetic cardiac inflammation and injury by activating the extracellular signal-regulated kinase (ERK)/early growth response-1 (Egr-1) pathway.^170^ Studies have shown that long-term exercise reduces the abundance of Gram-negative bacteria (e.g., *E. coli*) in the gut of patients with T2D and lowers circulating LPS levels.^97^^,^^98^ Therefore, exercise can confer cardioprotective benefits in diabetes by suppressing gut-derived LPS-induced myocardial inflammatory injury.

#### BCAAs

BCAAs are important metabolites produced by the gut microbiota, including leucine, isoleucine, and valine. In DCM mouse models, the abundance of BCAA-synthesizing taxa, such as *Clostridiales* and *Lachnospiraceae*, is increased, whereas BCAA-degrading species, including *Butyrivibrio* and *Eubacterium*, are reduced, leading to markedly elevated plasma BCAA levels.^81^^,^^171^ Excessive BCAAs suppress hepatic expression of peroxisome PPARα, leading to reduced secretion of FGF21. Consequently, this upregulates the myocardial amino acid transporter L-type amino acid transporter 1 (LAT1) and promotes the influx of BCAAs into the heart. The resulting accumulation of BCAAs in cardiomyocytes aberrantly activates the mTOR signaling pathway, causing mitochondrial injury and cardiomyocyte apoptosis, which ultimately contribute to myocardial fibrosis and functional impairment.^81^ Moreover, elevated BCAA levels—particularly leucine—activate the mTOR/S6 kinase (S6K) pathway, thereby impairing insulin signaling and promoting insulin resistance.^172^ Clinical evidence indicates that 12 weeks of high-intensity-combined aerobic and resistance exercise program increases BCAA-catabolizing genera such as *Eubacterium* and *Bacteroides*. This microbial shift reduces circulating BCAA levels and improves glucose homeostasis and insulin sensitivity.^99^ Collectively, these findings suggest that exercise can help prevent and treat DCM by regulating BCAA metabolism.

#### H_2_S

H_2_S is produced by host enzymatic pathways, including cystathionine γ-lyase and cystathionine β-synthase, as well as by gut microbial metabolism, particularly by sulfate-reducing bacteria such as *Desulfovibrio*.^173^^,^^174^ At physiological concentrations, H_2_S plays a critical role in maintaining cardiac homeostasis.^175^ In DCM models, however, impaired H_2_S biosynthesis reduces myocardial H_2_S availability, thereby exacerbated pathological cardiac remodeling.^176^^,^^177^ Extensive studies have demonstrated that exogenous H_2_S donors (e.g., NaHS) exert protective effects against DCM. For instance, exogenous H_2_S administration in STZ-induced diabetic rats prevents the inactivation of iron-sulfur proteins, improves mitochondrial function, and consequently alleviates myocardial injury.^178^ Moreover, H_2_S upregulates the stress-responsive protein Sestrin2 and inhibits the TLR4/MyD88/NF-κB pathway, thereby reducing inflammation and pyroptosis in cardiac fibroblasts under hyperglycemic conditions and ultimately mitigating cardiac fibrosis.^179^ Recent findings further suggest that, in diabetic mice, exogenous H_2_S attenuates myocardial necroptosis by suppressing cardiac NLRP3 inflammasome activation and enhancing E2F transcription factor 1 (E2F1)-mediated RAR-related orphan receptor α (RORα) transcription in cardiomyocytes, thereby protecting against DCM.^180^^,^^181^

Interestingly, 4 weeks of high-intensity aerobic exercise has been reported to selectively enrich *Desulfovibrio*, suggesting that exercise may enhance gut-derived H_2_S production and thereby contribute to improved cardiac outcomes in DCM.^182^ Nevertheless, direct evidence-linking exercise, gut-derived H_2_S, and DCM outcomes remains limited. To date, no study has conclusively shown that exercise increases gut-derived H_2_S, either locally within the gut or systemically in circulation. Moreover, the sources of H_2_S within the heart are difficult to distinguish, and gut-derived H_2_S is rapidly metabolized, further complicating its assessment.^183^^,^^184^ Thus, this proposed mechanism requires further validation.

#### Tryptophan metabolites

Dietary tryptophan is metabolized by the gut microbiota into indole and its derivatives, including indole-3-acetic acid (IAA) and indole-3-propionic acid (IPA), which play important roles in regulating host metabolism and immune function. In the myocardium, IPA binds to the aryl hydrocarbon receptor (AhR) and upregulates sirtuin-3 (SIRT3) expression, thereby restoring the nicotinamide adenine dinucleotide (NAD^+^) salvage pathway and improving the metabolic and diastolic dysfunction associated with heart failure with preserved ejection fraction (HFpEF).^185^ Likewise, IAA suppresses activation of the TLR4/myeloid differentiation factor 88 (MyD88)/NF-κB signaling pathway in M1 macrophages and promotes M2 polarization, thereby mitigating cardiovascular inflammation.^186^ Studies have shown that aerobic exercise increases the abundance of indole-producing bacteria, such as *A. muciniphila* and *Clostridium*, resulting in elevated levels of IPA and IAA in both serum and intestinal contents.^187^ Thus, exercise may improve DCM by enhancing the production of these indole derivatives, although this remains to be confirmed.

In conclusion, exercise plays an important role in the prevention and treatment of DCM by modulating gut microbiota-derived metabolites. It should be noted that the metabolites discussed previously are not exclusively derived from the gut microbiota. Certain metabolites, including BCAAs, H_2_S, and IAA, can also originate from host metabolic pathways. For example, cardiomyocytes and other host cells can produce endogenous H_2_S via enzymatic pathways involving cystathionine β-synthase, cystathionine γ-lyase, and 3-mercaptopyruvate sulfurtransferase.^188^ BCAAs are released during host protein turnover and amino acid catabolism,^189^ whereas IAA can be generated through minor host-dependent tryptophan-indoleamine metabolic bypass pathways, albeit at relatively low levels.^190^ Therefore, future studies should employ microbiota-validation approaches, such as antibiotic-mediated microbiota depletion and FMT, in combination with targeted interrogation of host metabolic pathways (e.g., inhibition of key host enzymes), to disentangle microbial versus host contributions and quantitatively assess host-derived inputs. In parallel, stable isotope-tracing strategies integrated with multi-omics analyses (metagenomics, metabolomics, transcriptomics, and proteomics) should be applied to map metabolic fluxes, link specific microbial taxa or functional genes to metabolite production, and connect these pathways to downstream cardiac signaling responses that underlie exercise-mediated protection against DCM. In addition, the interaction between exercise, gut microbiota-derived metabolites, and cardiac function is not unidirectional; rather, bidirectional communication and feedback loops exist.^191^^,^^192^ Improvements in diabetic cardiac dysfunction mediated by microbial metabolites can enhance intestinal perfusion, autonomic nervous system activity, and intestinal barrier integrity, which in turn further promote gut microbial homeostasis.^193^^,^^194^^,^^195^ Thus, exercise functions not only as an upstream regulator of gut microbiota-derived metabolites but also as a dynamic component of an adaptive feedback system. Recognizing these interactions may provide a more comprehensive framework for understanding the exercise-gut-heart axis and highlight the need for longitudinal and systems-level studies to disentangle causal relationships within this axis in DCM.

### Improvement of intestinal barrier function

The intestinal barrier constitutes the first line of host immune defense, serving a pivotal role in preventing the entry of harmful toxins and microorganisms while maintaining efficient nutrient absorption. It is composed mainly of a physical barrier formed by a single layer of epithelial cells linked by tight junction proteins (TJs), including occludin, claudin, and ZO-1, along with a chemical barrier provided by the overlying mucus layer.^196^ TJs determine whether substances can pass through transcellular or paracellular pathways from the mucosal to the serosal side, thereby regulating intestinal permeability.^197^

As previously described, elevated intestinal LPS levels and reduced SCFA concentrations in patients with diabetes impair gut barrier function.^169^^,^^198^ Moreover, chronic heart failure decreases cardiac output, causing intestinal ischemia and hypoxia that further disrupt epithelial integrity.^199^ These factors collectively increase intestinal permeability, a condition commonly known as “leaky gut”. The leaky gut condition facilitates the translocation of LPS and pathogenic bacteria into the circulation, triggering systemic low-grade inflammation and metabolic disturbances, thereby accelerating the progression of DCM.^15^

Exercise-induced modulation of the gut microbiota plays a critical role in enhancing intestinal barrier function. Pasini et al. reported that 6 months of combined aerobic, resistance, and flexibility training suppressed the overgrowth of intestinal fungi, including *Candida albicans* and *Mycetes* spp., in patients with T2D. The reduction of these fungi attenuates intestinal immune-inflammatory responses, thereby mitigating epithelial injury and limiting the translocation of LPS into the circulation.^98^ Exercise-induced microbial shifts also reduce the formation of neutrophil extracellular traps (NETs) and diminish intestinal immune-cell infiltration, further supporting the preservation of barrier integrity.^200^ Moreover, regular exercise increases the abundance of specific beneficial bacteria in patients with T2D, including *A*. *muciniphila*, *Bacteroides dorei*, and *Bacteroides vulgatus*.^94^^,^^97^ These taxa participate in preserving epithelial cohesion and limiting metabolic endotoxemia. For example, the outer-membrane protein Amuc_1100 from *A*. *muciniphila* enhances the expression of occludin and Tj protein-1 (Tjp-1), thereby strengthening intestinal barrier integrity.^201^ In addition, *A. muciniphila* stimulates the secretion of endogenous endocannabinoids in the gut, which exert anti-inflammatory effects and help maintain barrier function.^202^ *Bacteroides vulgatus* and *Bacteroides dorei* upregulate colonic Tjs gene expression, resulting in reduced intestinal permeability, decreased luminal LPS production, and attenuation of endotoxemia.^203^

**Figure 2:**
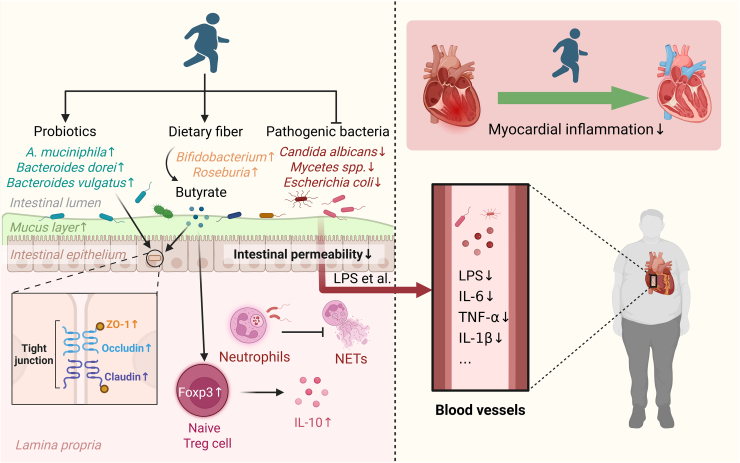
Mechanism of exercise enhances intestinal barrier function prevent and treat DCM Regular exercise suppresses the overgrowth of intestinal fungi (e.g., *Candida albicans*, *Mycetes* spp.) and decreases NET formation, thereby reducing mucosal immune-inflammatory responses and limiting LPS translocation into the circulation. Exercise also increases the abundance of beneficial bacteria, such as *A*. *muciniphila*, *Bacteroides dorei*, and *Bacteroides vulgatus*, which promote tight-junction protein expression, preserve epithelial cohesion, and attenuate metabolic endotoxemia. Additionally, exercise elevates gut butyrate production, which stimulates epithelial proliferation, strengthens tight-junction assembly, and induces colonic Treg cell differentiation to support anti-inflammatory signaling. Together, these adaptations enhance intestinal barrier function and reduce systemic inflammation, indirectly contributing to cardioprotection in diabetes. ZO-1, zonula occludens-1. Created in BioRender. Chen, J. (2025) https://BioRender.com/7g0c616.

Beyond these bacterial shifts, exercise also enhances gut butyrate production in patients with T2D.^94^ Butyrate has been shown to promote the proliferation and differentiation of intestinal epithelial cells and upregulate the expression of TJs to repair and strengthen intercellular connections.^204^ It also induces colonic regulatory T (Treg) cell differentiation by upregulating forkhead box P3 (Foxp3) expression and stimulates the production of anti-inflammatory cytokines, such as IL-10, thereby maintaining intestinal epithelial barrier function.^205^ Collectively, these findings suggest that regular exercise strengthens intestinal barrier integrity and alleviates systemic inflammation, thereby indirectly exerting cardioprotective effects in patients with diabetes (Figure 2).

Nevertheless, caution is warranted regarding exercise intensity. Evidence indicates that in healthy individuals, high-intensity aerobic exercise (80% VO_2max_) induces a markedly greater increase in gut permeability than moderate- or low-intensity exercise (40%–60% VO_2max_).^206^ In obese men, HIIT (100% maximal aerobic speed) results in significantly higher circulating LPS levels than MICT (65% maximal aerobic speed),^207^ potentially due to HIIT-induced enrichment of microbial taxa associated with increased gut leakage.^208^ Therefore, in patients with diabetes—particularly those with DCM—excessive high-intensity exercise should be avoided.

### Conclusions and future prospects

The gut-heart axis represents a critical pathophysiological and therapeutic interface linking gut microbiota dysbiosis, metabolic disturbances, and diabetes-related cardiac injury. Exercise-induced alterations in the gut microbiota may contribute to the prevention and amelioration of DCM through this axis.

Despite growing insights into the exercise-gut-heart axis, several important gaps remain. First, most existing studies have focused on T2D, whereas evidence in T1D remains scarce. Given the unique autoimmune pathogenesis and absolute insulin deficiency in T1D, future research should determine whether comparable exercise-induced microbial adaptations occur and how these affect cardiac outcomes. Second, establishing causal relationships between specific microbial taxa and DCM pathogenesis is essential for identifying microbial drivers of disease progression. Such insights would facilitate the development of precision exercise prescriptions aimed at effectively modulating these microbes. Moreover, accumulating evidence suggests that inter-individual variability in exercise responsiveness be partly determined by baseline gut microbiota composition and functional capacity.^99^^,^^209^^,^^210^ Therefore, incorporating baseline gut microbiome and metabolite profiling help identify exercise “responders” and “non-responders” and enable stratified exercise interventions. Future studies should combine baseline microbiota characterization with standardized exercise protocols and longitudinal outcome assessment to establish predictive microbiome signatures of exercise efficacy in DCM. Furthermore, most current mechanistic insights are derived from diabetic animal models or associative observational studies in humans. In diabetes and DCM, direct evidence linking exercise-induced alterations in the gut microbiota and specific microbial metabolites (such as H_2_S) to cardiac outcomes remains scarce. Furthermore, the human gut microbial metabolome is highly diverse.^211^ Other metabolites, such as 3-hydroxyphenylacetic acid, 4-hydroxybenzoic acid, also contribute to improved cardiac function and are modulated by exercise.^212^ Therefore, future studies should employ integrated multi-omics approaches—including metagenomics, metabolomics, transcriptomics, and proteomics—to comprehensively characterize interactions among exercise, the gut microbiota and their metabolites, and DCM pathology by simultaneously analyzing microbial composition and function, metabolite profiles, and cardiac tissue signaling pathways. Based on these mechanistic insights, it may also be possible to develop “exercise mimetics” that specifically target these gut microbial pathways. For example, administering selected microbial metabolites (or their analogs) could replicate the microbiota-derived effects of exercise and thereby confer protective benefits against DCM.

In addition, the gut microbiota is strongly influenced by dietary factors. Several studies indicate that exercise combined with dietary interventions, such as high-fiber or low-carbohydrate diets, provides additional benefits in improving gut microbial composition.^209^^,^^213^^,^^214^ Thus, exercise alone may not represent the optimal strategy for preventing and treating DCM, and future research should explore how best to optimize combined exercise-diet interventions. This may include incorporating gut microbiome and metabolite profiling into the disease-risk assessment of patients with DCM, thereby enabling the development of personalized exercise-diet interventions. For example, patients with more elevated circulating TMAO may benefit more from reducing dietary intake of choline- and carnitine-rich foods during exercise interventions; those with more elevated LPS levels may respond better to exercise-diet interventions aimed at reducing gut permeability, such as moderate-intensity aerobic training, combined with a high-fiber diet; whereas patients with excessive microbial production of BCAAs may require strict restriction of BCAA-rich foods without altering total protein intake. Ultimately, clinicians may use this information to design personalized exercise-diet intervention strategies that maximize improvements in cardiac health among patients with diabetes.

Finally, emerging evidence suggests that the gut microbiota regulates cancer and chronic diseases not only through metabolic signaling but also by remodeling the intestinal and systemic microenvironment, including immune modulation and host-microbiota-drug interactions.^215^^,^^216^^,^^217^ Notably, commonly used antidiabetic drugs, such as metformin and sodium-glucose cotransporter 2 inhibitors, exert part of their therapeutic effects by modulating the gut microbiota and intestinal microenvironment.^15^ Within this broader framework, exercise may act as a multidimensional microenvironmental modulator, simultaneously shaping microbial composition, metabolite profiles, immune signaling, and host responsiveness to pharmacological therapies. Integrating these perspectives may provide a more comprehensive understanding of how exercise improves diabetes and its cardiovascular complications via the gut-heart axis.

In summary, exercise provides a safe and promising approach to modulating the gut microbiota for DCM prevention and treatment. Further elucidation of how exercise reshapes the gut microbiota and their metabolites to modulate the gut-heart axis will open new avenues for developing microbiota-directed interventions in DCM.

### Data and code availability

No new datasets, materials, or software were generated, as this work is a narrative review.

## Acknowledgments

This work was supported by the Macao Polytechnic University Research Project (grant No.: RP/FCSD-02/2025).

## Author contributions

J.C. was responsible for the overall conceptual design, literature collection, and manuscript writing. Z.F. and Y.L. were responsible for revising, polishing, and proofreading the entire manuscript. B.G. and R.D. contributed to the overall organization and structural layout of the article. All authors read and approved the final manuscript.

## Declaration of interests

The authors declare no commercial or financial relationships that could be construed as a potential conflict of interest.

## References

[1] Sun H., Saeedi P., Karuranga S., Pinkepank M., Ogurtsova K., Duncan B.B., Stein C., Basit A., Chan J.C.N., Mbanya J.C. (2022). IDF Diabetes Atlas: Global, regional and country-level diabetes prevalence estimates for 2021 and projections for 2045. Diabetes Res. Clin. Pract..

[2] Rubler S., Dlugash J., Yuceoglu Y.Z., Kumral T., Branwood A.W., Grishman A. (1972). New type of cardiomyopathy associated with diabetic glomerulosclerosis. Am. J. Cardiol..

[3] Konduracka E., Cieslik G., Galicka-Latala D., Rostoff P., Pietrucha A., Latacz P., Gajos G., Malecki M.T., Nessler J. (2013). Myocardial dysfunction and chronic heart failure in patients with long-lasting type 1 diabetes: a 7-year prospective cohort study. Acta Diabetol..

[4] Rizza V., Tondi L., Patti A.M., Cecchi D., Lombardi M., Perone F., Ambrosetti M., Rizzo M., Cianflone D., Maranta F. (2024). Diabetic cardiomyopathy: pathophysiology, imaging assessment and therapeutical strategies. Int. J. Cardiol. Cardiovasc. Risk Prev..

[5] Seferović P.M., Paulus W.J., Rosano G., Polovina M., Petrie M.C., Jhund P.S., Tschöpe C., Sattar N., Piepoli M., Papp Z. (2024). Diabetic myocardial disorder. A clinical consensus statement of the Heart Failure Association of the ESC and the ESC Working Group on Myocardial & Pericardial Diseases. Eur. J. Heart Fail..

[6] Tan Y., Zhang Z., Zheng C., Wintergerst K.A., Keller B.B., Cai L. (2020). Mechanisms of diabetic cardiomyopathy and potential therapeutic strategies: preclinical and clinical evidence. Nat. Rev. Cardiol..

[7] Zhong L., Hou X., Tian Y., Fu X. (2025). Exercise and dietary interventions in the management of diabetic cardiomyopathy: mechanisms and implications. Cardiovasc. Diabetol..

[8] Cassidy S., Thoma C., Hallsworth K., Parikh J., Hollingsworth K.G., Taylor R., Jakovljevic D.G., Trenell M.I. (2016). High intensity intermittent exercise improves cardiac structure and function and reduces liver fat in patients with type 2 diabetes: a randomised controlled trial. Diabetologia.

[9] Stolen K.Q., Kemppainen J., Kalliokoski K.K., Luotolahti M., Viljanen T., Nuutila P., Knuuti J. (2003). Exercise training improves insulin-stimulated myocardial glucose uptake in patients with dilated cardiomyopathy. J. Nucl. Cardiol..

[10] Loganathan R., Bilgen M., Al-Hafez B., Zhero S.V., Alenezy M.D., Smirnova I.V. (2007). Exercise training improves cardiac performance in diabetes: in vivo demonstration with quantitative cine-MRI analyses. J. Appl. Physiol..

[11] Echouffo-Tcheugui J.B., Perreault L., Ji L., Dagogo-Jack S. (2023). Diagnosis and Management of Prediabetes: A Review. JAMA.

[12] Peña Carrillo B.J., Sivasengh R., Johnstone A.M., Gabriel B.M. (2024). Exercise, nutrition and medicine timing in metabolic health: implications for management of type 2 diabetes. Proc Nutr Soc.

[13] Chen X., Zhang H., Ren S., Ding Y., Remex N.S., Bhuiyan M.S., Qu J., Tang X. (2023). Gut microbiota and microbiota-derived metabolites in cardiovascular diseases. Chin. Med. J..

[14] Mostafavi Abdolmaleky H., Zhou J.R. (2024). Gut Microbiota Dysbiosis, Oxidative Stress, Inflammation, and Epigenetic Alterations in Metabolic Diseases. Antioxidants.

[15] Jin J.Y., Yang X.Y., Feng R., Ye M.L., Xu H., Wang J.Y., Hu J.C., Zuo H.T., Lu J.Y., Song J.Y. (2025). Gut Microbiota-Derived Metabolites Orchestrate Metabolic Reprogramming in Diabetic Cardiomyopathy: Mechanisms and Therapeutic Frontiers. Faseb J.

[16] Snelson M., R Muralitharan R., Liu C.F., Markó L., Forslund S.K., Marques F.Z., Tang W.H.W. (2025). Gut-Heart Axis: The Role of Gut Microbiota and Metabolites in Heart Failure. Circ. Res..

[17] Tsai H.J., Tsai W.C., Hung W.C., Hung W.W., Chang C.C., Dai C.Y., Tsai Y.C. (2021). Gut Microbiota and Subclinical Cardiovascular Disease in Patients with Type 2 Diabetes Mellitus. Nutrients.

[18] Cui X., Ye L., Li J., Jin L., Wang W., Li S., Bao M., Wu S., Li L., Geng B. (2018). Metagenomic and metabolomic analyses unveil dysbiosis of gut microbiota in chronic heart failure patients. Sci. Rep..

[19] Singhal S., Rani V. (2024). Therapeutic Potential of Syzygium aromaticum in Gut Dysbiosis via TMAO Associated Diabetic Cardiomyopathy. Cardiovasc. Hematol. Agents Med. Chem..

[20] Mohr A.E., Mach N., Pugh J., Grosicki G.J., Allen J.M., Karl J.P., Whisner C.M. (2025). Mechanisms underlying alterations of the gut microbiota by exercise and their role in shaping ecological resilience. FEMS Microbiol. Rev..

[21] Valder S., Brinkmann C. (2022). Exercise for the Diabetic Gut-Potential Health Effects and Underlying Mechanisms. Nutrients.

[22] Xie S.Y., Liu S.Q., Zhang T., Shi W.K., Xing Y., Fang W.X., Zhang M., Chen M.Y., Xu S.C., Fan M.Q. (2024). USP28 Serves as a Key Suppressor of Mitochondrial Morphofunctional Defects and Cardiac Dysfunction in the Diabetic Heart. Circulation.

[23] Delbridge L.M.D., Mellor K.M., Taylor D.J., Gottlieb R.A. (2017). Myocardial stress and autophagy: mechanisms and potential therapies. Nat. Rev. Cardiol..

[24] Montaigne D., Marechal X., Coisne A., Debry N., Modine T., Fayad G., Potelle C., El Arid J.M., Mouton S., Sebti Y. (2014). Myocardial contractile dysfunction is associated with impaired mitochondrial function and dynamics in type 2 diabetic but not in obese patients. Circulation.

[25] Kanamori H., Takemura G., Goto K., Tsujimoto A., Mikami A., Ogino A., Watanabe T., Morishita K., Okada H., Kawasaki M. (2015). Autophagic adaptations in diabetic cardiomyopathy differ between type 1 and type 2 diabetes. Autophagy.

[26] Dillmann W.H. (2019). Diabetic Cardiomyopathy. Circ. Res..

[27] Bellemare M., Bourcier L., Iglesies-Grau J., Boulet J., O'Meara E., Bouabdallaoui N. (2025). Mechanisms of diabetic cardiomyopathy: Focus on inflammation. Diabetes Obes. Metab..

[28] Jin L., Geng L., Ying L., Shu L., Ye K., Yang R., Liu Y., Wang Y., Cai Y., Jiang X. (2022). FGF21-Sirtuin 3 Axis Confers the Protective Effects of Exercise Against Diabetic Cardiomyopathy by Governing Mitochondrial Integrity. Circulation.

[29] Aragno M., Mastrocola R., Medana C., Catalano M.G., Vercellinatto I., Danni O., Boccuzzi G. (2006). Oxidative stress-dependent impairment of cardiac-specific transcription factors in experimental diabetes. Endocrinology.

[30] Umbarawan Y., Kawakami R., Syamsunarno M.R.A.A., Obinata H., Yamaguchi A., Hanaoka H., Hishiki T., Hayakawa N., Koitabashi N., Sunaga H. (2021). Reduced Fatty Acid Use from CD36 Deficiency Deteriorates Streptozotocin-Induced Diabetic Cardiomyopathy in Mice. Metabolites.

[31] Law B.A., Liao X., Moore K.S., Southard A., Roddy P., Ji R., Szulc Z., Bielawska A., Schulze P.C., Cowart L.A. (2018). Lipotoxic very-long-chain ceramides cause mitochondrial dysfunction, oxidative stress, and cell death in cardiomyocytes. Faseb J.

[32] Avagimyan A., Popov S., Shalnova S. (2022). The Pathophysiological Basis of Diabetic Cardiomyopathy Development. Curr. Probl. Cardiol..

[33] Jimenez R.E., Kubli D.A., Gustafsson Å.B. (2014). Autophagy and mitophagy in the myocardium: therapeutic potential and concerns. Br. J. Pharmacol..

[34] Tong M., Saito T., Zhai P., Oka S.I., Mizushima W., Nakamura M., Ikeda S., Shirakabe A., Sadoshima J. (2019). Mitophagy Is Essential for Maintaining Cardiac Function During High Fat Diet-Induced Diabetic Cardiomyopathy. Circ. Res..

[35] Lee J., Lee S., Zhang H., Hill M.A., Zhang C., Park Y. (2017). Interaction of IL-6 and TNF-α contributes to endothelial dysfunction in type 2 diabetic mouse hearts. PLoS One.

[36] Knapp M., Tu X., Wu R. (2019). Vascular endothelial dysfunction, a major mediator in diabetic cardiomyopathy. Acta Pharmacol. Sin..

[37] Khanna S., Singh G.B., Khullar M. (2014). Nitric oxide synthases and diabetic cardiomyopathy. Nitric Oxide.

[38] Quan C., Du Q., Li M., Wang R., Ouyang Q., Su S., Zhu S., Chen Q., Sheng Y., Chen L. (2020). A PKB-SPEG signaling nexus links insulin resistance with diabetic cardiomyopathy by regulating calcium homeostasis. Nat. Commun..

[39] Liu P., Zhang Z., Chen H., Chen Q. (2024). Pyroptosis: Mechanisms and links with diabetic cardiomyopathy. Ageing Res. Rev..

[40] Li S., Shu Y., Yang S., Zhang S., Chen H., Wu D., Li B., Dong L. (2025). Oxidative stress and ferroptosis in diabetic cardiomyopathy: mechanistic interplay and therapeutic implications. Apoptosis.

[41] Dimitropoulos G., Tahrani A.A., Stevens M.J. (2014). Cardiac autonomic neuropathy in patients with diabetes mellitus. World J. Diabetes.

[42] Wu H., Zhang P., Zhou J., Hu S., Hao J., Zhong Z., Yu H., Yang J., Chi J., Guo H. (2024). Paeoniflorin confers ferroptosis resistance by regulating the gut microbiota and its metabolites in diabetic cardiomyopathy. Am. J. Physiol. Cell Physiol..

[43] Zhu J., Bao Z., Hu Z., Wu S., Tian C., Zhou Y., Ding Z., Tan X. (2024). Myricetin alleviates diabetic cardiomyopathy by regulating gut microbiota and their metabolites. Nutr. Diabetes.

[44] Khalaf E.M., Hassan H.M., El-Baz A.M., Shata A., Khodir A.E., Yousef M.E., Elgharabawy R.M., Nouh N.A., Saleh S., Bin-Meferij M.M. (2022). A novel therapeutic combination of dapagliflozin, Lactobacillus and crocin attenuates diabetic cardiomyopathy in rats: Role of oxidative stress, gut microbiota, and PPARγ activation. Eur. J. Pharmacol..

[45] Lozupone C.A., Stombaugh J.I., Gordon J.I., Jansson J.K., Knight R. (2012). Diversity, stability and resilience of the human gut microbiota. Nature.

[46] Ley R.E., Peterson D.A., Gordon J.I. (2006). Ecological and evolutionary forces shaping microbial diversity in the human intestine. Cell.

[47] Hsu C.L., Schnabl B. (2023). The gut-liver axis and gut microbiota in health and liver disease. Nat. Rev. Microbiol..

[48] Barreto H.C., Gordo I. (2023). Intrahost evolution of the gut microbiota. Nat. Rev. Microbiol..

[49] Donald K., Finlay B.B. (2023). Early-life interactions between the microbiota and immune system: impact on immune system development and atopic disease. Nat. Rev. Immunol..

[50] Stacy A., Andrade-Oliveira V., McCulloch J.A., Hild B., Oh J.H., Perez-Chaparro P.J., Sim C.K., Lim A.I., Link V.M., Enamorado M. (2021). Infection trains the host for microbiota-enhanced resistance to pathogens. Cell.

[51] Romaní-Pérez M., Líebana-García R., Flor-Duro A., Bonillo-Jiménez D., Bullich-Vilarrubias C., Olivares M., Sanz Y. (2025). Obesity and the gut microbiota: implications of neuroendocrine and immune signaling. FEBS J..

[52] Neuman H., Forsythe P., Uzan A., Avni O., Koren O. (2018). Antibiotics in early life: dysbiosis and the damage done. FEMS Microbiol. Rev..

[53] Fan L., Xia Y., Wang Y., Han D., Liu Y., Li J., Fu J., Wang L., Gan Z., Liu B. (2023). Gut microbiota bridges dietary nutrients and host immunity. Sci. China Life Sci..

[54] Wang W., Liang S., Cao H., Lyu Y., Zhao Q., Zhang Z., Yang W. (2025). Physical Activity, Gut Microbiota, and the Risk of Dyslipidemia in a Community-Based Cohort Study. J. Am. Heart Assoc..

[55] Vanuytsel T., Bercik P., Boeckxstaens G. (2023). Understanding neuroimmune interactions in disorders of gut-brain interaction: from functional to immune-mediated disorders. Gut.

[56] Miyauchi E., Shimokawa C., Steimle A., Desai M.S., Ohno H. (2023). The impact of the gut microbiome on extra-intestinal autoimmune diseases. Nat. Rev. Immunol..

[57] Gurung M., Li Z., You H., Rodrigues R., Jump D.B., Morgun A., Shulzhenko N. (2020). Role of gut microbiota in type 2 diabetes pathophysiology. EBioMedicine.

[58] Cani P.D., Van Hul M. (2024). Gut microbiota in overweight and obesity: crosstalk with adipose tissue. Nat. Rev. Gastroenterol. Hepatol..

[59] Yu J., Yang Y.N., Chen W., Hu J., Jin Z., Wu C., Li Y. (2025). Role of gut microbiota and derived metabolites in cardiovascular diseases. iScience.

[60] Fang X., Zhang Y., Huang X., Miao R., Zhang Y., Tian J. (2025). Gut microbiome research: Revealing the pathological mechanisms and treatment strategies of type 2 diabetes. Diabetes Obes. Metab..

[61] Dey P., Dey P. (2025). Mechanisms and implications of the gut microbial modulation of intestinal metabolic processes. NPJ Metab. Health Dis..

[62] Chen Q., Xue Y., Song X., Zhu B. (2019). Characteristics of gut microbiota in patients with diabetes mellitus and diabetic cardiovascular complications. Acta Microbiol. Sin..

[63] Deng X., Zhang C., Wang P., Wei W., Shi X., Wang P., Yang J., Wang L., Tang S., Fang Y. (2022). Cardiovascular Benefits of Empagliflozin Are Associated With Gut Microbiota and Plasma Metabolites in Type 2 Diabetes. J. Clin. Endocrinol. Metab..

[64] Li Z., Xing J., Ma X., Zhang W., Wang C., Wang Y., Qi X., Liu Y., Jian D., Cheng X. (2024). An orally administered bacterial membrane protein nanodrug ameliorates doxorubicin cardiotoxicity through alleviating impaired intestinal barrier. Bioact. Mater..

[65] Gou H.Z., Zhang Y.L., Ren L.F., Li Z.J., Zhang L. (2022). How do intestinal probiotics restore the intestinal barrier?. Front. Microbiol..

[66] Zhou Y., Yang T., Zheng S., Gan T., Yu F., Liu G., Zhou T. (2025). Genetical TRPV4 deletion-associated gut microbiota alleviates cardiac dysfunction in mice with diabetic cardiomyopathy. J. Mol. Cell. Cardiol..

[67] Wan J., Zhang J., Xu Q., Yin H., Chen D., Yu B., He J. (2021). Alginate oligosaccharide protects against enterotoxigenic Escherichia coli-induced porcine intestinal barrier injury. Carbohydr. Polym..

[68] Yuan M., Sun T., Zhang Y., Guo C., Wang F., Yao Z., Yu L. (2024). Quercetin Alleviates Insulin Resistance and Repairs Intestinal Barrier in db/db Mice by Modulating Gut Microbiota. Nutrients.

[69] Tulkens J., Vergauwen G., Van Deun J., Geeurickx E., Dhondt B., Lippens L., De Scheerder M.A., Miinalainen I., Rappu P., De Geest B.G. (2020). Increased levels of systemic LPS-positive bacterial extracellular vesicles in patients with intestinal barrier dysfunction. Gut.

[70] Zhang C., Teng X., Cao Q., Deng Y., Yang M., Wang L., Rui D., Ling X., Wei C., Chen Y. (2025). Gut microbiota dysbiosis exacerbates heart failure by the LPS-TLR4/NF-κB signalling axis: mechanistic insights and therapeutic potential of TLR4 inhibition. J. Transl. Med..

[71] Yang J., Song J., Zhou J., Lin H., Wu Z., Liu N., Xie W., Guo H., Chi J. (2022). Functional components of Chinese rice wine can ameliorate diabetic cardiomyopathy through the modulation of autophagy, apoptosis, gut microbiota, and metabolites. Front. Cardiovasc. Med..

[72] Huang Y.L., Xiang Q., Zou J.J., Wu Y., Yu R. (2023). Zuogui Jiangtang Shuxin formula Ameliorates diabetic cardiomyopathy mice via modulating gut-heart axis. Front. Endocrinol..

[73] Fang C., Xu X., Lu F., Liu S. (2025). Study on the collaborative protective mechanism of Scutellariae Radix and Paeoniae Radix Alba against diabetic cardiomyopathy through the gut-heart axis. Front. Microbiol..

[74] Yue S., Zhao D., Peng C., Tan C., Wang Q., Gong J. (2019). Effects of theabrownin on serum metabolites and gut microbiome in rats with a high-sugar diet. Food Funct..

[75] Mazanko M.S., Mazanko E.V., Emelyantsev S.A., Chikindas M.L., Rudoy D.V. (2025). Lactobacilli and gut pH: features of correlation in model experiments. Braz. J. Microbiol..

[76] DeSana A.J., Estus S., Barrett T.A., Saatman K.E. (2024). Acute gastrointestinal permeability after traumatic brain injury in mice precedes a bloom in Akkermansia muciniphila supported by intestinal hypoxia. Sci. Rep..

[77] Feng J., Qian Y., Zhou Z., Ertmer S., Vivas E.I., Lan F., Hamilton J.J., Rey F.E., Anantharaman K., Venturelli O.S. (2022). Polysaccharide utilization loci in Bacteroides determine population fitness and community-level interactions. Cell Host Microbe.

[78] Schwalm N.D., Groisman E.A. (2017). Navigating the Gut Buffet: Control of Polysaccharide Utilization in Bacteroides spp. Trends Microbiol..

[79] Bastin M., Andreelli F. (2020). The gut microbiota and diabetic cardiomyopathy in humans. Diabetes Metab..

[80] Zhao Z., Hu Z., Li L. (2025). Cardiac energy metabolic disorder and gut microbiota imbalance: a study on the therapeutic potential of Shenfu Injection in rats with heart failure. Front. Microbiol..

[81] Zheng H., Zhang X., Li C., Wang D., Shen Y., Lu J., Zhao L., Li X., Gao H. (2024). BCAA mediated microbiota-liver-heart crosstalk regulates diabetic cardiomyopathy via FGF21. Microbiome.

[82] Wang H., Wang J., Cui H., Fan C., Xue Y., Liu H., Li H., Li J., Li H., Sun Y. (2024). Inhibition of fatty acid uptake by TGR5 prevents diabetic cardiomyopathy. Nat. Metab..

[83] Wu H., Liu G., He Y., Da J., Xie B. (2019). Obeticholic acid protects against diabetic cardiomyopathy by activation of FXR/Nrf2 signaling in db/db mice. Eur. J. Pharmacol..

[84] Forslund K., Hildebrand F., Nielsen T., Falony G., Le Chatelier E., Sunagawa S., Prifti E., Vieira-Silva S., Gudmundsdottir V., Pedersen H.K. (2015). Disentangling type 2 diabetes and metformin treatment signatures in the human gut microbiota. Nature.

[85] Lee D.M., Battson M.L., Jarrell D.K., Hou S., Ecton K.E., Weir T.L., Gentile C.L. (2018). SGLT2 inhibition via dapagliflozin improves generalized vascular dysfunction and alters the gut microbiota in type 2 diabetic mice. Cardiovasc. Diabetol..

[86] Rodrigues A., Gonçalves A., Morais J., Araujo R., Falcão-Pires I. (2023). Diet-Induced Microbiome's Impact on Heart Failure: A Double-Edged Sword. Nutrients.

[87] Prakoso D., De Blasio M.J., Tate M., Ritchie R.H. (2022). Current landscape of preclinical models of diabetic cardiomyopathy. Trends Pharmacol. Sci..

[88] Riehle C., Bauersachs J. (2018). Of mice and men: models and mechanisms of diabetic cardiomyopathy. Basic Res. Cardiol..

[89] Lee W.S., Kim J. (2021). Application of Animal Models in Diabetic Cardiomyopathy. Diabetes Metab. J..

[90] Allen J.M., Mailing L.J., Cohrs J., Salmonson C., Fryer J.D., Nehra V., Hale V.L., Kashyap P., White B.A., Woods J.A. (2018). Exercise training-induced modification of the gut microbiota persists after microbiota colonization and attenuates the response to chemically-induced colitis in gnotobiotic mice. Gut Microbes.

[91] Clarke S.F., Murphy E.F., O'Sullivan O., Lucey A.J., Humphreys M., Hogan A., Hayes P., O'Reilly M., Jeffery I.B., Wood-Martin R. (2014). Exercise and associated dietary extremes impact on gut microbial diversity. Gut.

[92] Hawley J.A., Forster S.C., Giles E.M. (2025). Exercise, the Gut Microbiome and Gastrointestinal Diseases: Therapeutic Impact and Molecular Mechanisms. Gastroenterology.

[93] Lin W., Pu L., Qian X., Pan J., Cheng R., Sun P. (2025). Exercise-induced modulation of gut microbiota in individuals with obesity and type 2 diabetes: a systematic review and meta-analysis. Front. Microbiol..

[94] Torquati L., Gajanand T., Cox E.R., Willis C.R.G., Zaugg J., Keating S.E., Coombes J.S. (2023). Effects of exercise intensity on gut microbiome composition and function in people with type 2 diabetes. Eur. J. Sport Sci..

[95] Lambert J.E., Myslicki J.P., Bomhof M.R., Belke D.D., Shearer J., Reimer R.A. (2015). Exercise training modifies gut microbiota in normal and diabetic mice. Applied physiology, nutrition, and metabolism = Physiologie appliquee, nutrition et metabolisme.

[96] Shalon D., Culver R.N., Grembi J.A., Folz J., Treit P.V., Shi H., Rosenberger F.A., Dethlefsen L., Meng X., Yaffe E. (2023). Profiling the human intestinal environment under physiological conditions. Nature.

[97] Motiani K.K., Collado M.C., Eskelinen J.J., Virtanen K.A., Löyttyniemi E., Salminen S., Nuutila P., Kalliokoski K.K., Hannukainen J.C. (2020). Exercise Training Modulates Gut Microbiota Profile and Improves Endotoxemia. Med. Sci. Sports Exerc..

[98] Pasini E., Corsetti G., Assanelli D., Testa C., Romano C., Dioguardi F.S., Aquilani R. (2019). Effects of chronic exercise on gut microbiota and intestinal barrier in human with type 2 diabetes. Minerva Med..

[99] Liu Y., Wang Y., Ni Y., Cheung C.K.Y., Lam K.S.L., Wang Y., Xia Z., Ye D., Guo J., Tse M.A. (2020). Gut Microbiome Fermentation Determines the Efficacy of Exercise for Diabetes Prevention. Cell Metab..

[100] Yang L., Lin H., Lin W., Xu X. (2020). Exercise Ameliorates Insulin Resistance of Type 2 Diabetes through Motivating Short-Chain Fatty Acid-Mediated Skeletal Muscle Cell Autophagy. Biology.

[101] Han Y., Quan H., Ji W., Tian Q., Liu X., Liu W. (2024). Moderate-intensity continuous training and high-intensity interval training alleviate glycolipid metabolism through modulation of gut microbiota and their metabolite SCFAs in diabetic rats. Biochem. Biophys. Res. Commun..

[102] Allen J.M., Mailing L.J., Niemiro G.M., Moore R., Cook M.D., White B.A., Holscher H.D., Woods J.A. (2018). Exercise Alters Gut Microbiota Composition and Function in Lean and Obese Humans. Med. Sci. Sports Exerc..

[103] Suryani D., Subhan Alfaqih M., Gunadi J.W., Sylviana N., Goenawan H., Megantara I., Lesmana R. (2022). Type, Intensity, and Duration of Exercise as Regulator of Gut Microbiome Profile. Curr. Sports Med. Rep..

[104] Vandeputte D., Falony G., Vieira-Silva S., Tito R.Y., Joossens M., Raes J. (2016). Stool consistency is strongly associated with gut microbiota richness and composition, enterotypes and bacterial growth rates. Gut.

[105] Louis P., Flint H.J. (2017). Formation of propionate and butyrate by the human colonic microbiota. Environ. Microbiol..

[106] Litvak Y., Byndloss M.X., Tsolis R.M., Bäumler A.J. (2017). Dysbiotic Proteobacteria expansion: a microbial signature of epithelial dysfunction. Curr. Opin. Microbiol..

[107] Khaledi M., Darvishi M., Sameni F., Shahrjerdi S., Karami E., Barahui N., Hemmati J., Hasheminasab M.S., Sanae M.-J., Akhavan-Sigari R., Owlia P. (2024). Association between exercise and changes in gut microbiota profile. a review.

[108] Sasaki H., Miyakawa H., Watanabe A., Tamura K., Shiga K., Lyu Y., Ichikawa N., Fu Y., Hayashi K., Imamura M., Shibata S. (2022). Evening rather than morning increased physical activity alters the microbiota in mice and is associated with increased body temperature and sympathetic nervous system activation. Biochim. Biophys. Acta. Mol. Basis Dis..

[109] Wang G., Si Q., Yang S., Jiao T., Zhu H., Tian P., Wang L., Li X., Gong L., Zhao J. (2020). Lactic acid bacteria reduce diabetes symptoms in mice by alleviating gut microbiota dysbiosis and inflammation in different manners. Food Funct..

[110] Tang H., Huang W., Yao Y.F. (2023). The metabolites of lactic acid bacteria: classification, biosynthesis and modulation of gut microbiota. Microb. Cell.

[111] Jang L.G., Choi G., Kim S.W., Kim B.Y., Lee S., Park H. (2019). The combination of sport and sport-specific diet is associated with characteristics of gut microbiota: an observational study. J. Int. Soc. Sports Nutr..

[112] Boytar A.N., Skinner T.L., Wallen R.E., Jenkins D.G., Dekker Nitert M. (2023). The Effect of Exercise Prescription on the Human Gut Microbiota and Comparison between Clinical and Apparently Healthy Populations: A Systematic Review. Nutrients.

[113] Grosicki G.J., Durk R.P., Bagley J.R. (2019). Rapid gut microbiome changes in a world-class ultramarathon runner. Physiol. Rep..

[114] Sato M., Suzuki Y. (2022). Alterations in intestinal microbiota in ultramarathon runners. Sci. Rep..

[115] Henningsen K., Gaskell S.K., Young P., Mika A., Henry R., Costa R.J.S. (2025). The Impact of Ultra-Marathon Running on the Gut Microbiota as Determined by Faecal Bacterial Profiling, and Its Relationship with Exercise-Associated Gastrointestinal Symptoms: An Exploratory Investigation. Nutrients.

[116] McFadzean R.J.U.H.T. (2014).

[117] Evans C.C., LePard K.J., Kwak J.W., Stancukas M.C., Laskowski S., Dougherty J., Moulton L., Glawe A., Wang Y., Leone V. (2014). Exercise prevents weight gain and alters the gut microbiota in a mouse model of high fat diet-induced obesity. PLoS One.

[118] Cronin O., Barton W., Skuse P., Penney N.C., Garcia-Perez I., Murphy E.F., Woods T., Nugent H., Fanning A., Melgar S. (2018). A Prospective Metagenomic and Metabolomic Analysis of the Impact of Exercise and/or Whey Protein Supplementation on the Gut Microbiome of Sedentary Adults. mSystems.

[119] Mukhopadhya I., Louis P. (2025). Gut microbiota-derived short-chain fatty acids and their role in human health and disease. Nat. Rev. Microbiol..

[120] Tsai H.J., Tsai W.C., Yu P.S., Hung W.C., Hung W.W., Tsai Y.C. (2025). Circulating short-chain fatty acids and subclinical cardiovascular disease in type 2 diabetes mellitus. Pol Arch Intern Med *null*. Pol. Arch. Intern. Med..

[121] Shi F., Chen J. (2025). Exercise-Induced Short-Chain Fatty Acids: A Novel Therapeutic Target in Type 2 Diabetes Mellitus with Sarcopenia. Aging Dis.

[122] Tolhurst G., Heffron H., Lam Y.S., Parker H.E., Habib A.M., Diakogiannaki E., Cameron J., Grosse J., Reimann F., Gribble F.M. (2012). Short-chain fatty acids stimulate glucagon-like peptide-1 secretion via the G-protein-coupled receptor FFAR2. Diabetes.

[123] Salazar J., Angarita L., Morillo V., Navarro C., Martínez M.S., Chacín M., Torres W., Rajotia A., Rojas M., Cano C. (2020). Microbiota and Diabetes Mellitus: Role of Lipid Mediators. Nutrients.

[124] Sharma A.X., Quittner-Strom E.B., Lee Y., Johnson J.A., Martin S.A., Yu X., Li J., Lu J., Cai Z., Chen S. (2018). Glucagon Receptor Antagonism Improves Glucose Metabolism and Cardiac Function by Promoting AMP-Mediated Protein Kinase in Diabetic Mice. Cell Rep..

[125] Nogal A., Valdes A.M., Menni C. (2021). The role of short-chain fatty acids in the interplay between gut microbiota and diet in cardio-metabolic health. Gut Microbes.

[126] Lee M., Katerelos M., Gleich K., Galic S., Kemp B.E., Mount P.F., Power D.A. (2018). Phosphorylation of Acetyl-CoA Carboxylase by AMPK Reduces Renal Fibrosis and Is Essential for the Anti-Fibrotic Effect of Metformin. J. Am. Soc. Nephrol..

[127] Timmermans A.D., Balteau M., Gélinas R., Renguet E., Ginion A., de Meester C., Sakamoto K., Balligand J.L., Bontemps F., Vanoverschelde J.L. (2014). A-769662 potentiates the effect of other AMP-activated protein kinase activators on cardiac glucose uptake. Am. J. Physiol. Heart Circ. Physiol..

[128] Luiken J.J.F.P., Glatz J.F.C., Neumann D. (2015). Cardiac contraction-induced GLUT4 translocation requires dual signaling input. Trends Endocrinol. Metab..

[129] Chang W.T., Cheng J.T., Chen Z.C. (2016). Telmisartan improves cardiac fibrosis in diabetes through peroxisome proliferator activated receptor δ (PPARδ): from bedside to bench. Cardiovasc. Diabetol..

[130] Sun B., Jia Y., Hong J., Sun Q., Gao S., Hu Y., Zhao N., Zhao R. (2018). Sodium Butyrate Ameliorates High-Fat-Diet-Induced Non-alcoholic Fatty Liver Disease through Peroxisome Proliferator-Activated Receptor α-Mediated Activation of β Oxidation and Suppression of Inflammation. J. Agric. Food Chem..

[131] Challa A.A., Lewandowski E.D. (2022). Short-Chain Carbon Sources: Exploiting Pleiotropic Effects for Heart Failure Therapy. JACC, Basic Transl. Sci..

[132] Carley A.N., Maurya S.K., Fasano M., Wang Y., Selzman C.H., Drakos S.G., Lewandowski E.D. (2021). Short-Chain Fatty Acids Outpace Ketone Oxidation in the Failing Heart. Circulation.

[133] Panagia M., He H., Baka T., Pimentel D.R., Croteau D., Bachschmid M.M., Balschi J.A., Colucci W.S., Luptak I. (2020). Increasing mitochondrial ATP synthesis with butyrate normalizes ADP and contractile function in metabolic heart disease. NMR Biomed..

[134] Chen Y., Du J., Zhao Y.T., Zhang L., Lv G., Zhuang S., Qin G., Zhao T.C. (2015). Histone deacetylase (HDAC) inhibition improves myocardial function and prevents cardiac remodeling in diabetic mice. Cardiovasc. Diabetol..

[135] Guo Y., Zou J., Xu X., Zhou H., Sun X., Wu L., Zhang S., Zhong X., Xiong Z., Lin Y. (2021). Short-chain fatty acids combined with intronic DNA methylation of HIF3A: Potential predictors for diabetic cardiomyopathy. Clin. Nutr..

[136] Fang J., Yan W., Sun X., Chen J. (2025). The role of exercise-induced short-chain fatty acids in the gut-muscle axis: implications for sarcopenia prevention and therapy. Front. Microbiol..

[137] Martin-Gallausiaux C., Marinelli L., Blottière H.M., Larraufie P., Lapaque N. (2021). SCFA: mechanisms and functional importance in the gut. Proc. Nutr. Soc..

[138] Vasavan T., Ferraro E., Ibrahim E., Dixon P., Gorelik J., Williamson C. (2018). Heart and bile acids - Clinical consequences of altered bile acid metabolism. Biochim. Biophys. Acta. Mol. Basis Dis..

[139] Cai J., Rimal B., Jiang C., Chiang J.Y.L., Patterson A.D. (2022). Bile acid metabolism and signaling, the microbiota, and metabolic disease. Pharmacol. Ther..

[140] Poland J.C., Flynn C.R. (2021). Bile Acids, Their Receptors, and the Gut Microbiota. Physiology.

[141] Aoi W., Inoue R., Mizushima K., Honda A., Björnholm M., Takagi T., Naito Y. (2023). Exercise-acclimated microbiota improves skeletal muscle metabolism via circulating bile acid deconjugation. iScience.

[142] Carbajo-Pescador S., Porras D., García-Mediavilla M.V., Martínez-Flórez S., Juarez-Fernández M., Cuevas M.J., Mauriz J.L., González-Gallego J., Nistal E., Sánchez-Campos S. (2019). Beneficial effects of exercise on gut microbiota functionality and barrier integrity, and gut-liver crosstalk in an in vivo model of early obesity and non-alcoholic fatty liver disease. Dis. Model. Mech..

[143] Morville T., Sahl R.E., Trammell S.A., Svenningsen J.S., Gillum M.P., Helge J.W., Clemmensen C. (2018). Divergent effects of resistance and endurance exercise on plasma bile acids, FGF19, and FGF21 in humans. JCI Insight.

[144] Shi J., Cui J., Zheng T., Han X., Wang B., Wang W., Zhu C., Fang C., Zhou X., Cong N. (2025). Comparative effects of aerobic and resistance exercise on bile acid profiles and liver function in patients with non-alcoholic fatty liver disease. BMC Gastroenterol..

[145] Hui S., Huang L., Wang X., Zhu X., Zhou M., Chen M., Yi L., Mi M. (2020). Capsaicin improves glucose homeostasis by enhancing glucagon-like peptide-1 secretion through the regulation of bile acid metabolism via the remodeling of the gut microbiota in male mice. Faseb j.

[146] Brighton C.A., Rievaj J., Kuhre R.E., Glass L.L., Schoonjans K., Holst J.J., Gribble F.M., Reimann F. (2015). Bile Acids Trigger GLP-1 Release Predominantly by Accessing Basolaterally Located G Protein-Coupled Bile Acid Receptors. Endocrinology.

[147] Chiang J.Y.L., Ferrell J.M. (2020). Up to date on cholesterol 7 alpha-hydroxylase (CYP7A1) in bile acid synthesis. Liver Res..

[148] Zhang M., Xiao B., Chen X., Ou B., Wang S. (2024). Physical exercise plays a role in rebalancing the bile acids of enterohepatic axis in non-alcoholic fatty liver disease. Acta Physiol..

[149] Ngo Sock E.T., Farahnak Z., Lavoie J.M. (2014). Exercise training decreases gene expression of endo- and xeno-sensors in rat small intestine. Appl. Physiol. Nutr. Metabol..

[150] Qiang S., Tao L., Zhou J., Wang Q., Wang K., Lu M., Wang W., Han L., Xue S., Chen Y. (2020). Knockout of farnesoid X receptor aggravates process of diabetic cardiomyopathy. Diabetes Res. Clin. Pract..

[151] Wang J., Zhang J., Lin X., Wang Y., Wu X., Yang F., Gao W., Zhang Y., Sun J., Jiang C., Xu M. (2021). DCA-TGR5 signaling activation alleviates inflammatory response and improves cardiac function in myocardial infarction. J. Mol. Cell. Cardiol..

[152] Sousa Fialho M.D.L., Purnama U., Dennis K.M.J.H., Montes Aparicio C.N., Castro-Guarda M., Massourides E., Tyler D.J., Carr C.A., Heather L.C. (2021). Activation of HIF1α Rescues the Hypoxic Response and Reverses Metabolic Dysfunction in the Diabetic Heart. Diabetes.

[153] Zhu W., Gregory J.C., Org E., Buffa J.A., Gupta N., Wang Z., Li L., Fu X., Wu Y., Mehrabian M. (2016). Gut Microbial Metabolite TMAO Enhances Platelet Hyperreactivity and Thrombosis Risk. Cell.

[154] Zhang Y., Wang Y., Ke B., Du J. (2021). TMAO: how gut microbiota contributes to heart failure. Transl. Res..

[155] Croyal M., Saulnier P.J., Aguesse A., Gand E., Ragot S., Roussel R., Halimi J.M., Ducrocq G., Cariou B., Montaigne D. (2020). Plasma Trimethylamine N-Oxide and Risk of Cardiovascular Events in Patients With Type 2 Diabetes. J. Clin. Endocrinol. Metab..

[156] Yu N., Gu N., Wang Y., Zhou B., Lu D., Li J., Ma X., Zhang J., Guo X. (2022). The Association of Plasma Trimethylamine N-Oxide with Coronary Atherosclerotic Burden in Patients with Type 2 Diabetes Among a Chinese North Population. Diabetes Metab. Syndr. Obes..

[157] Flores-Guerrero J.L., van Dijk P.R., Connelly M.A., Garcia E., Bilo H.J.G., Navis G., Bakker S.J.L., Dullaart R.P.F. (2021). Circulating Trimethylamine N-Oxide Is Associated with Increased Risk of Cardiovascular Mortality in Type-2 Diabetes: Results from a Dutch Diabetes Cohort (ZODIAC-59). J. Clin. Med..

[158] Zhen J., Zhou Z., He M., Han H.X., Lv E.H., Wen P.B., Liu X., Wang Y.T., Cai X.C., Tian J.Q. (2023). The gut microbial metabolite trimethylamine N-oxide and cardiovascular diseases. Front. Endocrinol..

[159] Li X., Geng J., Zhao J., Ni Q., Zhao C., Zheng Y., Chen X., Wang L. (2019). Trimethylamine N-Oxide Exacerbates Cardiac Fibrosis via Activating the NLRP3 Inflammasome. Front. Physiol..

[160] Battiprolu P.K., Hojayev B., Jiang N., Wang Z.V., Luo X., Iglewski M., Shelton J.M., Gerard R.D., Rothermel B.A., Gillette T.G. (2012). Metabolic stress-induced activation of FoxO1 triggers diabetic cardiomyopathy in mice. J. Clin. Investig..

[161] Zhang X., Tang N., Hadden T.J., Rishi A.K. (2011). Akt, FoxO and regulation of apoptosis. Biochim. Biophys. Acta.

[162] Savi M., Bocchi L., Bresciani L., Falco A., Quaini F., Mena P., Brighenti F., Crozier A., Stilli D., Del Rio D. (2018). Trimethylamine-N-Oxide (TMAO)-Induced Impairment of Cardiomyocyte Function and the Protective Role of Urolithin B-Glucuronide. Molecules.

[163] Makrecka-Kuka M., Volska K., Antone U., Vilskersts R., Grinberga S., Bandere D., Liepinsh E., Dambrova M. (2017). Trimethylamine N-oxide impairs pyruvate and fatty acid oxidation in cardiac mitochondria. Toxicol. Lett..

[164] Ke J., Pan J., Lin H., Gu J. (2023). Diabetic cardiomyopathy: a brief summary on lipid toxicity. ESC Heart Fail..

[165] Argyridou S., Bernieh D., Henson J., Edwardson C.L., Davies M.J., Khunti K., Suzuki T., Yates T. (2020). Associations between physical activity and trimethylamine N -oxide in those at risk of type 2 diabetes. BMJ Open Diabetes Res. Care.

[166] Zhang H., Meng J., Yu H. (2017). Trimethylamine N-oxide Supplementation Abolishes the Cardioprotective Effects of Voluntary Exercise in Mice Fed a Western Diet. Front. Physiol..

[167] Zou H., Gong L., Huang C., Lin D., Zhang Y. (2025). The Protective Mechanism of Moderate Intensity Continuous Training on TMAO-Induced Myocardial Injury Based on NMR Metabolomics. Int. J. Mol. Sci..

[168] Di Vincenzo F., Del Gaudio A., Petito V., Lopetuso L.R., Scaldaferri F. (2024). Gut microbiota, intestinal permeability, and systemic inflammation: a narrative review. Intern. Emerg. Med..

[169] Gao F., Zhang X., Xu Z., Zhang K., Quan F. (2024). Goat milk derived small extracellular vesicles ameliorate LPS-induced intestinal epithelial barrier dysfunction, oxidative stress, and apoptosis by inhibiting the MAPK signaling pathway. Food Funct..

[170] Wu Q., Zhao M., Li D., He X., Zang W. (2023). Cholinergic drugs reduce metabolic inflammation and diabetic myocardial injury by regulating the gut bacterial component lipopolysaccharide-induced ERK/Egr-1 pathway. Faseb J.

[171] Yang Y., Zhao M., He X., Wu Q., Li D.L., Zang W.J. (2021). Pyridostigmine Protects Against Diabetic Cardiomyopathy by Regulating Vagal Activity, Gut Microbiota, and Branched-Chain Amino Acid Catabolism in Diabetic Mice. Front. Pharmacol..

[172] Vanweert F., Schrauwen P., Phielix E. (2022). Role of branched-chain amino acid metabolism in the pathogenesis of obesity and type 2 diabetes-related metabolic disturbances BCAA metabolism in type 2 diabetes. Nutr. Diabetes.

[173] Gui D.D., Luo W., Yan B.J., Ren Z., Tang Z.H., Liu L.S., Zhang J.F., Jiang Z.S. (2021). Effects of gut microbiota on atherosclerosis through hydrogen sulfide. Eur. J. Pharmacol..

[174] Wu D., Tan B., Sun Y., Hu Q. (2022). Cystathionine γ lyase S-sulfhydrates Drp1 to ameliorate heart dysfunction. Redox Biol..

[175] Lu Q.B., Zhu X.X., Chen G., Su J.B., Zhao C.Y., Xu A.J., Bian J.S., Sun H.J. (2025). Role of Hydrogen Sulfide Regulation of Programmed Cell Death: Implications for Cardiovascular Diseases. Antioxid. Redox Signal..

[176] Brancaleone V., Roviezzo F., Vellecco V., De Gruttola L., Bucci M., Cirino G. (2008). Biosynthesis of H2S is impaired in non-obese diabetic (NOD) mice. Br. J. Pharmacol..

[177] Jin S., Pu S.X., Hou C.L., Ma F.F., Li N., Li X.H., Tan B., Tao B.B., Wang M.J., Zhu Y.C. (2015). Cardiac H2S Generation Is Reduced in Ageing Diabetic Mice. Oxid. Med. Cell. Longev..

[178] Wang M., Zhang S., Tian J., Yang F., Chen H., Bai S., Kang J., Pang K., Huang J., Dong M. (2025). Impaired Iron-Sulfur Cluster Synthesis Induces Mitochondrial PARthanatos in Diabetic Cardiomyopathy. Adv. Sci..

[179] Liu Y., Yang T., Hu H., Yang Q., Yang J., Chu C. (2025). Hydrogen sulfide (H 2 S) alleviates diabetic myocardial fibrosis by suppressing pyroptosis via inhibiting DNMT3a-mediated Sestrin2 CpG promoter hypermethylation. Arch. Biochem. Biophys..

[180] Gong W., Zhang S., Chen Y., Shen J., Zheng Y., Liu X., Zhu M., Meng G. (2022). Protective role of hydrogen sulfide against diabetic cardiomyopathy via alleviating necroptosis. Free Radic. Biol. Med..

[181] Zhang S., Shen J., Zhu Y., Zheng Y., San W., Cao D., Chen Y., Meng G. (2023). Hydrogen sulfide promoted retinoic acid-related orphan receptor α transcription to alleviate diabetic cardiomyopathy. Biochem. Pharmacol..

[182] Fernández J., Fernández-Sanjurjo M., Iglesias-Gutiérrez E., Martínez-Camblor P., Villar C.J., Tomás-Zapico C., Fernández-García B., Lombó F. (2021). Resistance and Endurance Exercise Training Induce Differential Changes in Gut Microbiota Composition in Murine Models. Front. Physiol..

[183] Powell C.R., Dillon K.M., Matson J.B. (2018). A review of hydrogen sulfide (H 2 S) donors: Chemistry and potential therapeutic applications. Biochem. Pharmacol..

[184] Birg A., Lin H.C. (2025). The Role of Bacteria-Derived Hydrogen Sulfide in Multiple Axes of Disease. Int. J. Mol. Sci..

[185] Wang Y.C., Koay Y.C., Pan C., Zhou Z., Tang W., Wilcox J., Li X.S., Zagouras A., Marques F., Allayee H. (2024). Indole-3-Propionic Acid Protects Against Heart Failure With Preserved Ejection Fraction. Circ. Res..

[186] Liu W., Wang J., Yang H., Li C., Lan W., Chen T., Tang Y. (2025). The Metabolite Indole-3-Acetic Acid of Bacteroides Ovatus Improves Atherosclerosis by Restoring the Polarisation Balance of M1/M2 Macrophages and Inhibiting Inflammation. Adv. Sci..

[187] Vazquez-Medina A., Rodriguez-Trujillo N., Ayuso-Rodriguez K., Marini-Martinez F., Angeli-Morales R., Caussade-Silvestrini G., Godoy-Vitorino F., Chorna N. (2024). Exploring the interplay between running exercises, microbial diversity, and tryptophan metabolism along the microbiota-gut-brain axis. Front. Microbiol..

[188] Strutynska N., Strutynskyi R., Mys L., Luchkova A., Korkach Y., Goshovska Y., Chorna S., Sagach V. (2022). Exercise restores endogenous H 2 S synthesis and mitochondrial function in the heart of old rats. Eur. J. Clin. Invest..

[189] Dimou A., Tsimihodimos V., Bairaktari E. (2022). The Critical Role of the Branched Chain Amino Acids (BCAAs) Catabolism-Regulating Enzymes, Branched-Chain Aminotransferase (BCAT) and Branched-Chain α-Keto Acid Dehydrogenase (BCKD). Int. J. Mol. Sci..

[190] Zhang X., Gan M., Li J., Li H., Su M., Tan D., Wang S., Jia M., Zhang L., Chen G. (2020). Endogenous Indole Pyruvate Pathway for Tryptophan Metabolism Mediated by IL4I1. J. Agric. Food Chem..

[191] Yun S., Seo Y., Lee Y., Lee D.T. (2024). Gut microbiome related to metabolic diseases after moderate-to-vigorous intensity exercise. J. Exerc. Sci. Fit..

[192] Guers J.J., Heffernan K.S., Campbell S.C. (2025). Getting to the Heart of the Matter: Exploring the Intersection of Cardiovascular Disease, Sex and Race and How Exercise, and Gut Microbiota Influence these Relationships. Rev. Cardiovasc. Med..

[193] Tang W.H.W., Li D.Y., Hazen S.L. (2019). Dietary metabolism, the gut microbiome, and heart failure. Nat. Rev. Cardiol..

[194] Sandek A., Bjarnason I., Volk H.D., Crane R., Meddings J.B., Niebauer J., Kalra P.R., Buhner S., Herrmann R., Springer J. (2012). Studies on bacterial endotoxin and intestinal absorption function in patients with chronic heart failure. Int. J. Cardiol..

[195] Calvillo L., Vanoli E., Ferrara F., Caradonna E. (2025). Interplay Among Gut Microbiota-Derived TMAO, Autonomic Nervous System Dysfunction, and Heart Failure Progression. Int. J. Mol. Sci..

[196] Ghosh S., Whitley C.S., Haribabu B., Jala V.R. (2021). Regulation of Intestinal Barrier Function by Microbial Metabolites. Cell. Mol. Gastroenterol. Hepatol..

[197] Chelakkot C., Ghim J., Ryu S.H. (2018). Mechanisms regulating intestinal barrier integrity and its pathological implications. Exp. Mol. Med..

[198] Snelson M., de Pasquale C., Ekinci E.I., Coughlan M.T. (2021). Gut microbiome, prebiotics, intestinal permeability and diabetes complications. Best Pract. Res. Clin. Endocrinol. Metab..

[199] Pasini E., Aquilani R., Testa C., Baiardi P., Angioletti S., Boschi F., Verri M., Dioguardi F. (2016). Pathogenic Gut Flora in Patients With Chronic Heart Failure. JACC. Heart Fail..

[200] Zhu B., Wu H., Zhang H., Song Q., Xiao Y., Yu B. (2025). Gut microbiota from voluntary exercised mice protects the intestinal barrier by inhibiting neutrophil extracellular trap formation. iScience.

[201] Plovier H., Everard A., Druart C., Depommier C., Van Hul M., Geurts L., Chilloux J., Ottman N., Duparc T., Lichtenstein L. (2017). A purified membrane protein from Akkermansia muciniphila or the pasteurized bacterium improves metabolism in obese and diabetic mice. Nat. Med..

[202] Everard A., Belzer C., Geurts L., Ouwerkerk J.P., Druart C., Bindels L.B., Guiot Y., Derrien M., Muccioli G.G., Delzenne N.M. (2013). Cross-talk between Akkermansia muciniphila and intestinal epithelium controls diet-induced obesity. Proc. Natl. Acad. Sci. USA.

[203] Yoshida N., Emoto T., Yamashita T., Watanabe H., Hayashi T., Tabata T., Hoshi N., Hatano N., Ozawa G., Sasaki N. (2018). Bacteroides vulgatus and Bacteroides dorei Reduce Gut Microbial Lipopolysaccharide Production and Inhibit Atherosclerosis. Circulation.

[204] Wang H.B., Wang P.Y., Wang X., Wan Y.L., Liu Y.C. (2012). Butyrate enhances intestinal epithelial barrier function via up-regulation of tight junction protein Claudin-1 transcription. Dig. Dis. Sci..

[205] Wang H., Shi P., Zuo L., Dong J., Zhao J., Liu Q., Zhu W. (2016). Dietary Non-digestible Polysaccharides Ameliorate Intestinal Epithelial Barrier Dysfunction in IL-10 Knockout Mice. J. Crohns Colitis.

[206] Pals K.L., Chang R.T., Ryan A.J., Gisolfi C.V. (1997). Effect of running intensity on intestinal permeability. J. Appl. Physiol..

[207] Gerosa-Neto J., Monteiro P.A., Inoue D.S., Antunes B.M., Batatinha H., Dorneles G.P., Peres A., Rosa-Neto J.C., Lira F.S. (2020). High- and moderate-intensity training modify LPS-induced ex-vivo interleukin-10 production in obese men in response to an acute exercise bout. Cytokine.

[208] Peng M., Zou R., Yao S., Meng X., Wu W., Zeng F., Chen Z., Yuan S., Zhao F., Liu W. (2024). High-intensity interval training and medium-intensity continuous training may affect cognitive function through regulation of intestinal microbial composition and its metabolite LPS by the gut-brain axis. Life Sci..

[209] Cheng R., Wang L., Le S., Yang Y., Zhao C., Zhang X., Yang X., Xu T., Xu L., Wiklund P. (2022). A randomized controlled trial for response of microbiome network to exercise and diet intervention in patients with nonalcoholic fatty liver disease. Nat. Commun..

[210] Mach N., Midoux C., Leclercq S., Pennarun S., Le Moyec L., Rué O., Robert C., Sallé G., Barrey E. (2022). Mining the equine gut metagenome: poorly-characterized taxa associated with cardiovascular fitness in endurance athletes. Commun. Biol..

[211] Kruger R., Oler E., Saha S., Poelzer J., Han S., Punsalan M.P., Green B., Bushra F., Kyes M., Disu F. (2026). MiMeDB 2.0: the Human Microbial Metabolome Database for 2026. Nucleic Acids Res *null*. Nucleic Acids Res..

[212] Zhou Q., Deng J., Pan X., Meng D., Zhu Y., Bai Y., Shi C., Duan Y., Wang T., Li X. (2022). Gut microbiome mediates the protective effects of exercise after myocardial infarction. Microbiome.

[213] Lin Z., Zhang X., Wu M., Ming Y., Wang X., Li H., Huang F., Gao F., Zhu Y. (2023). High-fiber diet and rope-skipping benefit cardiometabolic health and modulate gut microbiota in young adults: A randomized controlled trial. Food Res. Int..

[214] Zha Y., Xiang M., Zuo Y., Liu D., Wang Q. (2025). High-dose Dietary Fibre Supplementation Enhances the Gut Microbiome, Health, and Athletic Performance of College Basketball Players. Int. J. Vitam. Nutr. Res..

[215] Lin A., Xiong M., Jiang A., Huang L., Wong H.Z.H., Feng S., Zhang C., Li Y., Chen L., Chi H. (2025). The microbiome in cancer. iMeta.

[216] Chen J., Li M., Sun W., Chen Y., Sheng Y., Lu C., Liu Z. (2025). A Dual Action of Everolimus in Ulcerative Colitis: Blocking CLEC4E-Driven Inflammation and Remodeling the Gut Microbiota-Metabolite Axis. iMetaMed.

[217] Gao Z., Jiang A., Li Z., Zhu L., Mou W., Shen W., Luo P., Tang B., Zhang J., Lin A. (2025). Heterogeneity of intratumoral microbiota within the tumor microenvironment and relationship to tumor development. Med Res.

